# A Novel *Dhillonvirus* Phage against *Escherichia coli* Bearing a Unique Gene of Intergeneric Origin

**DOI:** 10.3390/cimb46090551

**Published:** 2024-08-23

**Authors:** Anastasios Vasileiadis, Petros Bozidis, Konstantinos Konstantinidis, Nikolaos Kesesidis, Louiza Potamiti, Anna Kolliopoulou, Apostolos Beloukas, Mihalis I. Panayiotidis, Sophia Havaki, Vassilis G. Gorgoulis, Konstantina Gartzonika, Ioannis Karakasiliotis

**Affiliations:** 1 Laboratory of Biology, Department of Medicine, Democritus University of Thrace, 68100 Alexandroupolis, Greece; anastasios.vasileiadis@yahoo.com (A.V.); kostaskons94@gmail.com (K.K.); nikokese2@mbg.duth.gr (N.K.); 2 Department of Microbiology, Faculty of Medicine, School of Health Sciences, University of Ioannina, 45332 Ioannina, Greece; pbozidis@uoi.gr (P.B.); kgartzon@uoi.gr (K.G.); 3 Department of Cancer Genetics, Therapeutics & Ultrastructural Pathology, The Cyprus Institute of Neurology & Genetics, Nicosia 2371, Cyprus; louizap@cing.ac.cy (L.P.); mihalisp@cing.ac.cy (M.I.P.); 4 Molecular Microbiology and Immunology Laboratory, Department of Biomedical Sciences, University of West Attica, 12243 Athens, Greece; akolliopoulou@uniwa.gr (A.K.); abeloukas@uniwa.gr (A.B.); 5 Molecular Carcinogenesis Group, Department of Histology and Embryology, Medical School, National and Kapodistrian University of Athens, 11527 Athens, Greece; shavaki@med.uoa.gr (S.H.); vgorg@med.uoa.gr (V.G.G.); 6 Biomedical Research Foundation, Academy of Athens, 11527 Athens, Greece; 7 Ninewells Hospital and Medical School, University of Dundee, Dundee DD1 9SY, UK; 8 Faculty Institute for Cancer Sciences, Manchester Academic Health Sciences Centre, University of Manchester, Manchester M20 4GJ, UK; 9 Faculty of Health and Medical Sciences, University of Surrey, Surrey GU2 7YH, UK

**Keywords:** Escherichia phage, whole-genome sequencing, *Dhillonvirus*, CDS28, phylogenetic analysis, recombination

## Abstract

Antibiotics resistance is expanding amongst pathogenic bacteria. Phage therapy is a revived concept for targeting bacteria with multiple antibiotics resistances. In the present study, we isolated and characterized a novel phage from hospital treatment plant input, using *Escherichia coli* (*E. coli*) as host bacterium. Phage lytic activity was detected by using soft agar assay. Whole-genome sequencing of the phage was performed by using Next-Generation Sequencing (NGS). Host range was determined using other species of bacteria and representative genogroups of *E. coli*. Whole-genome sequencing of the phage revealed that Escherichia phage Ioannina is a novel phage within the *Dhillonvirus* genus, but significantly diverged from other Dhillonviruses. Its genome is a 45,270 bp linear double-stranded DNA molecule that encodes 61 coding sequences (CDSs). The coding sequence of CDS28, a putative tail fiber protein, presented higher similarity to representatives of other phage families, signifying a possible recombination event. Escherichia phage Ioannina lytic activity was broad amongst the *E. coli* genogroups of clinical and environmental origin with multiple resistances. This phage may present in the future an important therapeutic tool against bacterial strains with multiple antibiotic resistances.

## 1. Introduction

Phages are viruses that can infect bacteria [1]. Bacteriophages are found everywhere throughout the environment (e.g., in oceans, drinking water and food we consume). They are found in large numbers, estimated to be approximately 10^31^ in total [2]. In addition, bacteriophages have a very important role in the regulation of the microbial balance in each ecosystem studied [3].

Due to their great diversity, bacteriophages have many niche applications in the food industry [4,5], biotechnology [6] and medicine [7]. In recent years, it has been shown that the use of bacteriophages in combination with antibiotics and disinfectants can break down biofilms and dramatically enhance the reduction of the bacteria load [8]. As the prevalence of antibiotic resistance is increasing worldwide, phage therapy is a promising alternative treatment modality [9,10]. Τhe importance of the identification of new phages is highlighted by the recent advances on the phage virome (phageome) and its balance with the host microbiome in patients and healthy individuals [11]. The dissection of the vast phage ecosystem showcased phages that infect not only pathogenic bacteria but also symbiotic commensal flora [12]. Such phages may regulate the abundance and function of important bacteria for mucosal homeostasis and metabolism [13]. 

*Escherichia coli* (*E. coli*), one of the most prevalent bacteria in the human gut, is a Gram-negative bacterium which belongs to the family of *Enterobacteriaceae* and plays an important role in the formation of the intestinal microbiome. *E. coli* strains are classified into more than 180 O-antigen serotypes [14]. Each serotype has distinct attributes and may differentially affect mucosal homeostasis [15]. ATCC 25922 is a strain of *E. coli* representative of serotype O6 and biotype 1 and has been characterized as part of the non-pathogenic symbiotic commensal flora. ATCC 25922 and other symbiotic bacteria may exert immunomodulatory effects on inflammatory conditions such as the allergic airway inflammation [16]. In addition, ATCC 25922 has been widely used as a reference strain in a plethora of quality control and for antibiotic susceptibility testing [17]. 

In the present study, we sought to isolate and characterize lytic bacteriophages against *E. coli* from raw sewage of a tertiary hospital, before biological treatment. A novel lytic bacteriophage belonging to a genetically distinct branch of Dhillonviruses was isolated and characterized both physically and genetically.

## 2. Materials and Methods

### 2.1. Bacterial Strains and Growth Conditions

Mueller–Hinton broth (MHB) (2 g/L of beef infusion solids, 1.5 g/L of starch and 17.5 g/L of casein hydrosylate) was used for the bacterial culture. *E. coli* 25922 (Becton Dickinson, France S.A.S.) strain was stored in MHB supplemented with 50% glycerol at −80 °C. *E. coli* 25922 was grown in MHB at 37 °C with vigorous rotary shaking at 250 rpm.

### 2.2. Sample Collections

Sewage wastewater samples were collected from the input of the wastewater treatment plant of the University Hospital of Ioannina, Epirus, Greece. Specifically, 16 samples were collected for a time span of 4 months (February–May) in 2018.

### 2.3. Phage Isolation and Enrichment

Totally, 60 mL of 6 different sewage samples were centrifuged at 1100× *g* for 15 min at room temperature, and the supernatant was filtered through a 0.2 μm membrane filter to remove bacterial debris. Phages were concentrated using the PEG method. Final concentrations of 10% PEG 8000 and 1 M of NaCl were added, and the filtered sample was incubated overnight at 4 °C for phage precipitation. The following day, the filtered sample was centrifuged at 3000× *g* for 1 h and 30 min at room temperature, and the pellet was resuspended in 2 mL of SM buffer (50 mM of Tris-Cl pH 7.5, MgSO_4_. 7 H_2_O 8 mM, NaCl 100 mM). Bacteriophage stocks were then stored at 4 °C. For phage enrichment, 100 μL of the bacteriophage stock was mixed with 1 mL of overnight-grown *E. coli* 25922 strain, 4 mL of medium culture (MHB) and 50 μL of 1 M CaCl_2_.

### 2.4. Bacteriophage Plaque Assay 

A total of 100 μL of bacteriophage enrichment stock was diluted into the MHB with serial 10-fold dilutions from 10^−1^ up to 10^−6^. Briefly, 100 μL of each dilution were mixed with 400 μL of the *E. coli* 25922 strain culture in the logarithmic growth phase (OD_600_nm = 0.4) and 35 μL of 1 Μ CaCl_2_. The mix was incubated at 37 °C for 8 min. Then, 3 mL of molten soft agar (0.8% agar and 1% MHB) was added and poured onto 1.5% Mueller–Hinton agar plates, and the plates were incubated overnight at 37 °C. The next day, plaques were observed and counted. 

### 2.5. Phage DNA Extraction

Single plaques were picked with a tip and inoculated in a mix of 1 mL of *E. coli* 25922 strain culture in the logarithmic growth phase, 2 mL of ΜHΒ and 30 μL of 1 Μ CaCl_2_. The enrichment was incubated overnight at 37 °C. Subsequently, chloroform was added on the enrichment culture at a final concentration of 0.2% (*v*/*v*). The sample was then centrifuged at 2000× *g* for 20 min at room temperature. Supernatant (1.8 mL) was treated with DNase I (1 μg/mL) and RNase A (12.5 μg/mL) at 37 °C for 30 min. Then, the phage enrichment was treated with 46 μL of 20% SDS and 18 μL of Proteinase K (10 mg/mL) and incubated at 56 °C for 30 min. Phenol-chloroform extraction and isopropanol precipitation using sodium acetate (3 M, pH 5.2) were used for DNA purification. Finally, DNA was resuspended in 50 μL 1% TE (Tris 10 mM, EDTA 1 mM, pH 7.4) and stored at −20 °C.

### 2.6. Genome Sequencing

Genomic DNA libraries were prepared by using an Ion Singleseq^TM^ 96 Kit (#A34763, ThermoFischer Scientific, CA, USA) according to the manufacturer’s instructions. The concentration of the library was measured using a Qubit^TM^ 4 Fluorometer (ThermoFischer Scientific, Eugene, OR, USA) and was loaded on an Ion 540^TM^ chip. Sequencing was performed on an Ion GeneStudio S5 System (ThermoFischer Scientific, CA, USA). The raw sequencing datasets for the current study are available in the NCBI Sequence Read Archive repository, under the Bioproject with accession number PRJNA941078 (NCBI BioProject database, https://www.ncbi.nlm.nih.gov/bioproject/PRJNA941078—last accessed date 13 March 2023).

### 2.7. Phage Genome Assembly and Characterization

Following the NGS procedure, quality trimmed reads were used as input for a de novo assembly using Trinity (v2.8.5) [18]. The generated contigs were aligned against the non-redundant (nr) nucleotide and protein databases by using BLASTn and BLASTx [19], respectively, in order to be annotated. Nucleotide sequences corresponding to the same BLAST hit were fed into the CAP3 tool [20], using default parameters, in order to generate assembly scaffolds. The assembled genomic sequence of the phage identified in this study is available in the NCBI Nucleotide repository (NCBI Nucleotide database, https://www.ncbi.nlm.nih.gov/nuccore—last, accessed date 13 March 2023) and can be accessed online using the GenBank accession number OQ589852. CDS analysis was carried out by using the SnapGene 6.0.2 tool (SnapGene, GSL Biotech LLC, Boston, USA). CDSs were individually annotated using the BLASTp tool [19]. The tRNAscan-SE v. 2.0 tool was used to find possible tRNA genes in the whole genome of the phage [21]. Genome organization analysis of the identified phage was performed via EasyFig v. 2.2.5 [22], including representative highly similar phages from the same taxon after BLASTn of the assembled genomic sequence.

### 2.8. Transmission Electron Microscopy (TEM) Methodology

The negative staining technique was applied for examining phages by TEM. Specifically, 5 μL of sample was allowed to be absorbed for 2 min to the surface of a Formvar/Carbon-coated copper grid. The used grids were placed previously in a glow discharge unit to render them hydrophilic. After absorption, each grid was blotted with a filter paper, washed thrice on drops of ultrapure water and placed on a drop of 2% aqueous uranyl acetate (UA) solution for 1 min. The excess UA was removed and the grids were left to air dry. Phages were then examined under a JEM 2100 Plus Transmission Electron Microscope (Jeol, Tokyo, Japan) operating at 120 kV and photographed with a Gatan OneView digital camera (Gatan, Inc., Pleasanton, CA, USA).

### 2.9. One-Step Growth Curve and Adsorption Assay

The latency period and burst size of isolated phage were determined by observing changes in the number of phage particles during a lytic cycle as described [23]. Briefly, host strain *E. coli* 25922 was grown at 37 °C until log phase (OD_600_ = 0.5, 10^8^ CFU/mL). Then, 990 μL of bacterial grown culture was mixed with 10 μL of phage suspension (10^8^ PFU/mL) to achieve a multiplicity of infection of 0.01. The mixture was incubated for 10 min at 37 °C and then centrifuged at 16,200× *g* for 5 min. The pellet was washed with 1 mL of MHB to remove the unabsorbed phages. This process was repeated once more, and the pellet was resuspended in 10 mL of MHB and incubated at 37 °C with shaking. Aliquots of 500 μL were collected at 10 min intervals for 2 h, and then in every sample was added 1% chloroform, followed by centrifugation at 13,800× *g* for 2 min. The supernatant was immediately diluted and plated by using the soft agar assay method for phage titers’ determination. The latent period was determined as the time between infection and the shortest incubation time, allowing a phage particle to reproduce inside an infected host cell. The burst size was calculated as the ratio between the number of phage particles produced during a lytic cycle and the initial infected bacterial cells. Experiments for one-step phage growth curve were carried out in triplicates.

Adsorption assay was performed to evaluate the efficiency of isolated phage to ab-sorb the host strain *E. coli* 25922. Briefly, 990 μL of log-phase culture (10^8^ CFU/mL) was mixed with 10 μL of diluted phage suspension (10^7^ PFU/mL), and the mixture was incubated at 37 °C for 10 min. Subsequently, the mixture was centrifuged at 16,200× *g* for 5 min, and the titer of the supernatant was estimated by using the soft agar assay method. The phage adsorption efficiency was determined with the equation (initial phage titer—residual phage titer in the supernatant/initial phage titer).

### 2.10. Thermal and pH Stability

To test the thermotolerance of the phage, 100 μL of a 10^−6^ dilution of the enrichment was tested using the soft agar assay method at 37, 40, 45, 50, 55 and 60 °C for 1 h in a thermal cycler. Τo test the stability of the phage, in different pH values (2, 4, 6, 8 and 10), sodium acetate (1 M) was prepared. A 100 μL quantity from the phage enrichment was mixed with 900 μL of sodium acetate (1 M) at each different pH value and incubated at 37 °C for 1 h. Afterwards, serial 10-fold dilutions were carried out, and 100 μL from dilution 10^−6^ was tested for phage titer determination using the soft agar assay method. Experiments for thermal and pH stability were carried out in triplicates.

### 2.11. Host Range Determination

For the host range determination, spot test was used to evaluate the lytic ability of the bacteriophage to form lytic plaques. The Escherichia phage Ioannina was tested against 47 *E. coli* isolates deriving from different environments (hospital waste water, wastewater treatment plant, river water), as well as clinical strains. Additionally, the phage isolate was tested against 9 reference strains and a clinical strain (*E. coli* O157:H7). Forty-seven *E. coli* isolates were representatives of all 4 phylogenetic groups (i.e., A, B1, B2 and D) according to Clermont’s schema and with a variety of AMR profile (from WT to MDR) [24,25]. Briefly, bacteria maintained as glycerol stocks at −80 °C were subcultured directly in Nutrient Agar (for pure isolates), or first in MacConkey Agar and subsequently in Nutrient Agar (to verify purity of the isolate). Bacterial colonies were used to prepare suspensions in 0.9% NaCl according to the MacFarland scale of 1–5. Then, suspensions were used for the spot assay, where bacteria were first spread on separate Nutrient Agar plates, and then 1 μL of the phage preparation (in LB, maintained at 4 °C) was added at the center of each plate. Spot assay results were evaluated on the next day. Phage lytic activity was shown by the appearance of visible lytic plaques at the site of enrichment application [26].

### 2.12. Phylogenetic Analysis

The phylogenetic analysis of the assembled phage was based on the amino acid sequences of three proteins, namely the major capsid protein, the large terminase subunit protein and the portal protein, which were used for the construction of the corresponding phylogenetic trees as in recently published literature [27,28,29,30]. Each amino acid sequence was used as input to BLASTp [19] against the non-redundant (nr) protein sequence database of NCBI with the “Max target sequences” parameter set to 1000. BLASTp hits with not less than 30% coverage and 30% identity were selected in order to download their corresponding and complete protein sequences in FASTA format. Taxonomy data of the phages that encoded the retrieved protein sequences were also fetched utilizing the NCBI tool Entrez-direct [31], and subsequently, the protein sequences were taxonomically filtered to include only a few phage entries as representatives from different taxa. All sequences were deposited to a FASTA formatted file and were successively input to MEGA11 software (v11.0.11) [32], for the elucidation of phylogenetic relationships. Lastly, the phylogenetic reconstruction was performed using the Maximum Likelihood (ML) method [33] for 100 bootstrap replications [34], and the inferred trees were exported via MEGA11. The same procedure, as described above, as well as alignments by MUSCLE (v3.8) [35] were performed for the phylogenetic analysis of a unique CDS28 (putative tail fiber protein). The VIRIDIC tool was used to calculate and visualize the isolated phage intergenomic relatedness using default parameters [36]. 

## 3. Results

In order to isolate and characterize lytic bacteriophages against *E. coli* ATCC 25922, sewage samples were collected from the Ioannina hospital wastewater treatment plant. Multiple passages of the inoculum were used for the enrichment of bacteriophages against *Ε. coli* ATCC 25922. Only samples that showed marked reduction in culture medium turbidity were further processed. Soft agar assays were carried out in order to assess the efficiency in plaque formation of bacteriophages, and single-plaque isolates were subsequently cultured on *E. coli* ATCC 25922.

### 3.1. Genome Characterization 

**Figure 1:**
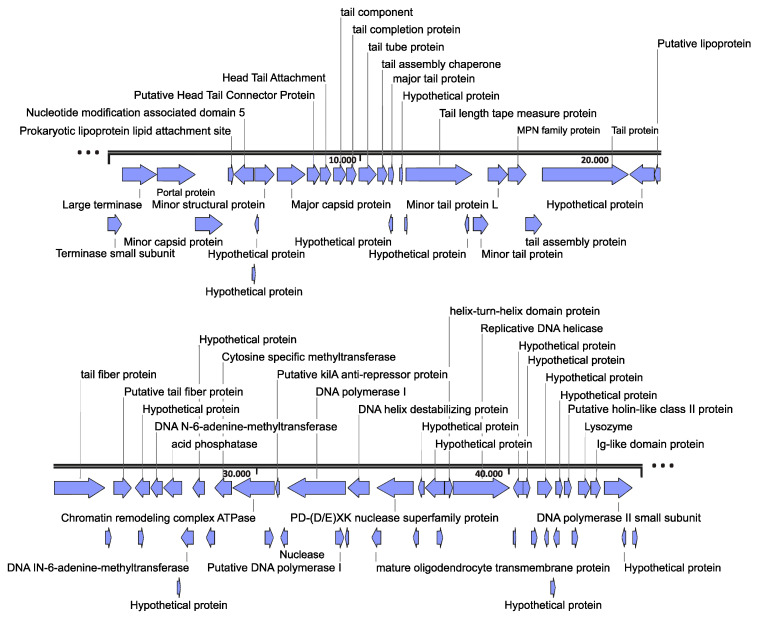
Map of the Escherichia phage Ioannina genome. Arrows with blue color represent predicted coding sequences (CDSs) of different phage functions. The genome map was constructed by using the SnapGene 6.0.2 tool.

Full-genome sequencing of the isolated bacteriophages was carried out using the Ion Torrent Next-Generation Sequencing (NGS) technology. The output NGS reads were assembled using the Trinity assembly (v2.8.5) software. The analysis showed that all samples yielded sequences of the same bacteriophage, named Escherichia phage Ioannina, which was a 45,270 bp linear double-stranded DNA molecule (Figure 1). The GC content of the identified bacteriophage was 53.62%. Using the SnapGene 6.0.2 tool, we predicted the presence of 61 putative coding sequences (CDSs), while no tRNA genes were found. Annotation of all predicted CDSs was performed by BLASTp tool, which indicated that 38 genes were on the forward and 23 were on the reverse strand (Figure 1, Supplementary Table S1). The nucleotide identity of the full-length genome compared to other closely related *Dhillonvirus* genomes reached up to 72.7% (Supplementary Table S2). Notably, the nucleotide identity of the full-length genome was closer (77.75%) to a phage metagenome (CtFRY1, NCBI GenBank accession number BK032676.1); however, no other details, besides the nucleotide sequence, could be retrieved for this GenBank entry.

### 3.2. Genome Organization

The CDS analysis (SnapGene 6.0.2) of the complete Escherichia phage Ioannina genome showed six distinct functional clusters, namely: (i) DNA replication, modification and transcriptional regulations: Replicative DNA helicase (CDS48), DNA polymerases (CDS39 and CDS60), DNA N-6-adenine methyltransferases (CDS30 and CDS32), Nucleotide modification-associated domain 5 (CDS6), Cytosine specific methyltransferase (CDS35), (ii) Head structure: Portal protein (CDS3), minor capsid protein (CDS4), minor structural protein (CDS7) and major capsid protein (CDS8), (iii) Packaging: DNA terminases (small subunit = CDS1 and large terminase = CDS2), mature oligodendrocyte transmembrane protein (CDS42), Putative Head Tail Connector Protein (CDS9) and Head Tail Attachment (CDS10), (iv) host lysis: Putative holin-like class II protein (CDS56), Putative holin-like class I protein (CDS57) and Lysozyme (CDS58), (v) tail structure: tail fiber proteins (CDS26 and CDS28), tail protein (CDS23), tail assembly proteins (CDS14 and CDS22), tail component (CDS11), tail completion protein (CDS12), tail tube protein (CDS13), major tail protein (CDS15), minor tail proteins (CDS19 and CDS20) and tail length tape measure protein (CDS18), (vi) hypothetical or unknown functions. A synteny plot of the Escherichia phage Ioannina genome against representative highly similar *Dhillonvirus* phages after BLASTn (Supplementary Table S2) showed that all these phages have nearly the same genome organization with the exception of some minor differences (Supplementary File S1). While all five phages seem to have the same genes encoding tail fiber proteins, one major difference is the presence of an additional putative tail protein (CDS28) on Escherichia phage Ioannina genome in contrast with the other representative *Dhillonvirus* phages.

### 3.3. Bacteriophage Plaque Formation and Morphology 

**Figure 2:**
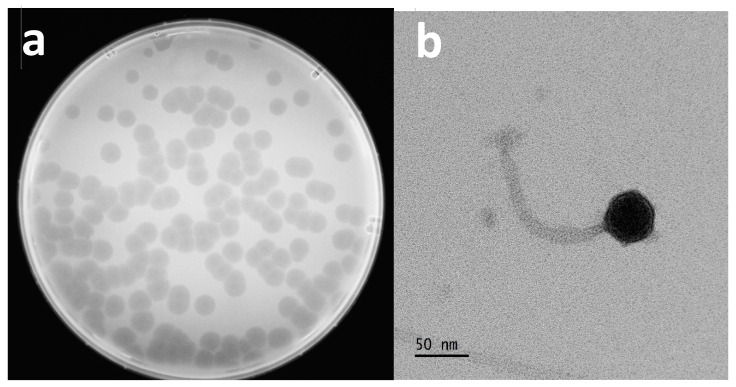
Escherichia phage Ioannina plaque morphology and morphology of the phage absorbed on the host. (**a**) Circular phage plaques with a diameter of about 5.3 ± 0.5 mm, formed on a bacterial lawn spread on MHB agar. (**b**) A TEM image of the phage was obtained, indicating a head and a non-contractile tail of 37 ± 3 nm and 122 ± 8 nm, respectively. UA negative staining. Scale bar represents 50 nm.

Following genomic characterization, we further analyzed the physical properties of the Escherichia phage Ioannina. By using the soft agar assay method, clear plaques of the bacteriophage were visible and uniform, showing a particularly potent lytic activity. The average size of the plaques was 5.3 ± 0.5 mm 16 h post infection (Figure 2a). 

Transmission electron microscopy (TEM) analysis showed that the Escherichia phage Ioannina consisted of a 37 ± 3 nm diameter icosahedral head and a long non-contractile tail 122 ± 8 nm long (Figure 2b). Based on the morphological characteristics, the phage could be classified as a siphovirus according to the International Committee on Taxonomy of Viruses (ICTV).

### 3.4. One-Step Growth Curve and Adsorption Assay

**Figure 3:**
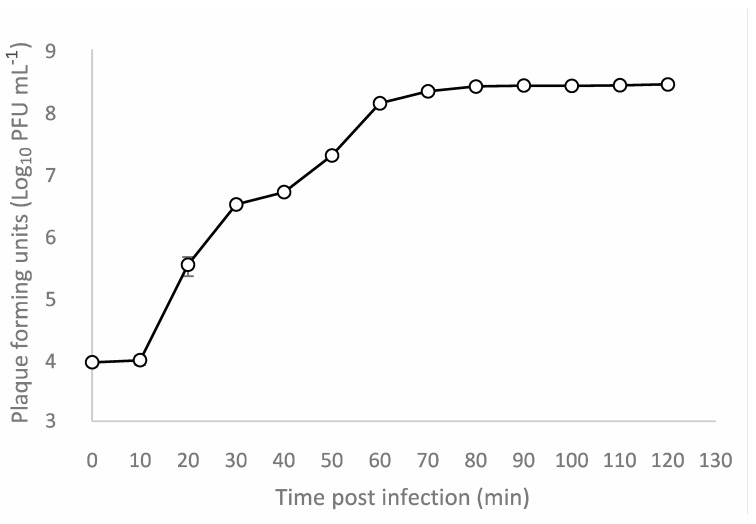
One-step growth curve of Escherichia phage Ioannina infecting *E. coli* ATCC 25922 strain at a multiplicity of infection of 0.01. Experiments for one-step phage growth curve were carried out in triplicates, and the standard deviations of the observed data are drawn as error bars on the depicted graph.

One-step growth experiment was performed to determine the latent period and the burst size of Escherichia phage Ioannina on host strain *E. coli* ATCC 25922. The latent period was 10 min, and the burst size was about 316 plaque-forming units (pfu) per infected cell (Figure 3). Adsorption efficiency of Escherichia phage Ioannina on host strain *E. coli* ATCC 25922 was approximately 99.83%.

### 3.5. Thermal and PH Stability of the Phage

**Figure 4:**
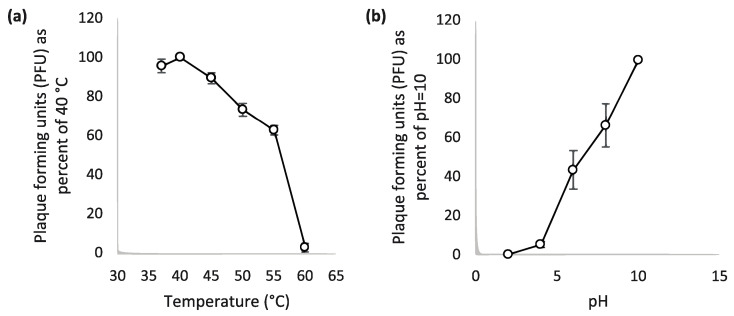
Thermal and pH stability analysis of Escherichia phage Ioannina. (**a**) The thermal stability of the phage after incubation at different temperatures for 1 h. (**b**) pH stability of the phage after incubation at different pH spectra for 1 h. Experiments for thermal and pH stability were carried out in triplicates, and the standard deviations of the observed data are drawn as error bars on the depicted graphs.

Temperature and pH are two important factors for the survival of a bacteriophage. For this reason, the stability of Escherichia phage Ioannina at different pH values (2, 4, 6, 8, 10) and temperatures (37, 40, 45, 50, 55, 60 °C) was determined (Figure 4a). Heat treatment for 1 h at 45–55 °C presented a gradual reduction of about 40% in phage viability as measured by its plaque-forming ability. Heat treatment at all temperatures greater than 40 °C diminished phage viability. Phage viability is eliminated at 60 °C. Τhe phage also showed highly lytic activity in alkaline pH. More specifically, the phage viability showed a constant reduction towards the acidic end of the pH spectrum (Figure 4b).

### 3.6. Host Range Determination

Host range determination was carried out by testing the identified phage against 47 *E. coli* isolates, nine reference strains and a clinical strain *E. coli* O157:H7. Host range testing revealed that Escherichia phage Ioannina was highly specific. The Escherichia phage Ioannina lysed the reference strain *E. coli* ATCC 25922, and 11 *E. coli* isolates derived from both environmental and clinical samples. Regarding the genotypes of *E. coli* strains susceptible to infection, they were found in all of four phylogenetic groups (A, B1, B2 and D). Escherichia phage Ioannina successfully infected several MDR *E. coli* clinical isolates. Further, Escherichia phage Ioannina could not infect the other tested *E. coli* strains and strains from other species. (Table 1).

### 3.7. Phylogenetic Analysis 

**Figure 5:**
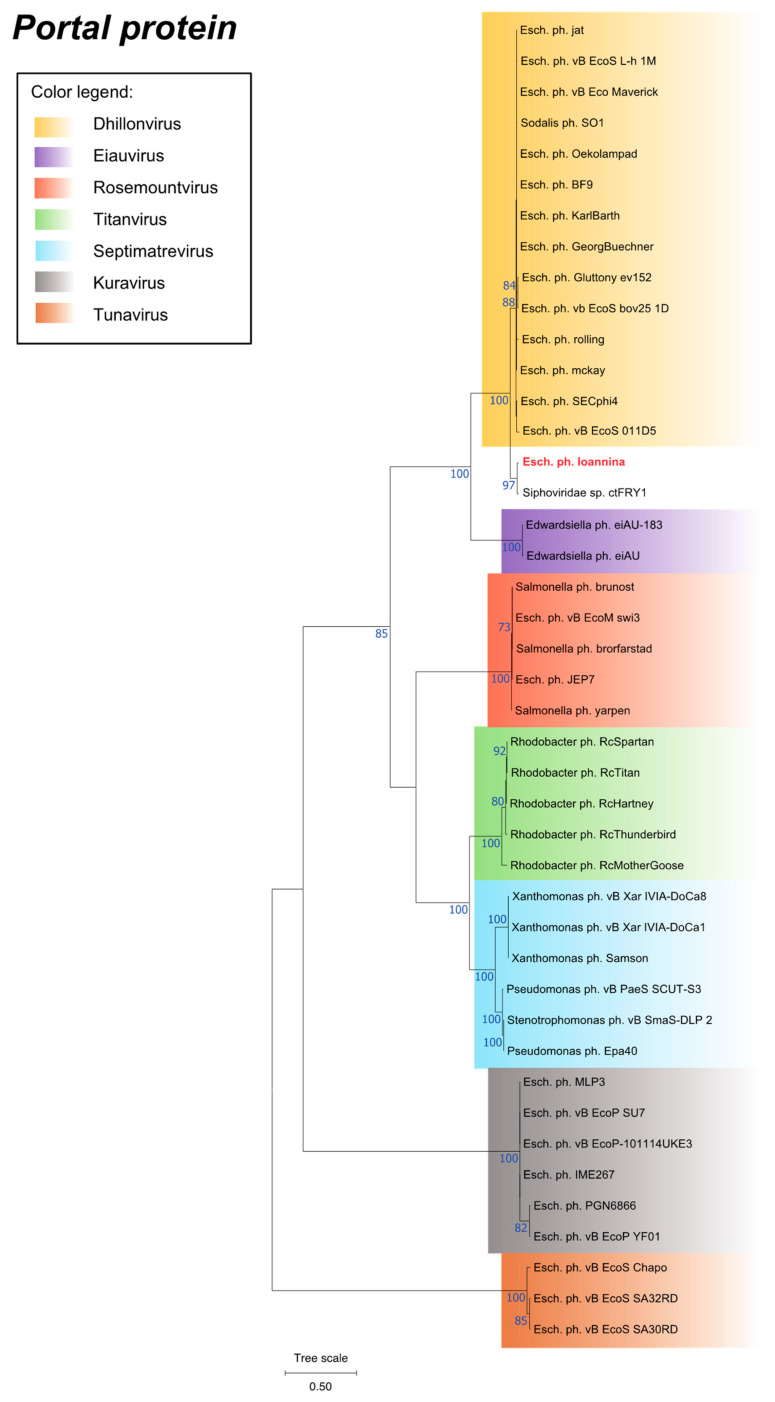
Phylogenetic tree of the Escherichia phage Ioannina portal protein constructed using the Maximum Likelihood method of the MEGA11 software. The “Esch. Ph. Ioannina” represents the Escherichia phage Ioannina portal protein. Bootstrap values (blue-colored text) were obtained from 100 bootstrap replicates, and only those above 70 are displayed next to each node. The tree scale is displayed on the bottom left corner of the phylogenetic tree.

Phylogenetic analysis was performed by utilizing the MEGA11 program and was mainly based on the portal protein of Escherichia phage Ioannina and other similar phages (Figure 5). Moreover, two additional proteins, namely the large terminase subunit protein and the major capsid protein, were used to verify the phylogeny of Escherichia phage Ioannina (Supplementary Figures S1 and S2). According to the phylogenetic analysis, Escherichia phage Ioannina can be classified as a siphovirus, forming a distinct cluster within the *Dhillonvirus* genus, which contained only the CtFRY1 metagenome (Figure 5, Supplementary Figures S1 and S2). The classification within the *Dhillonvirus* genus was further supported by calculation of the intergenomic similarities and distances amongst similar phages by utilizing the VIRIDIC tool (Supplementary Figure S3). 

### 3.8. Phylogenetic Analysis of a Unique Putative Tail Fiber Protein

**Figure 6:**
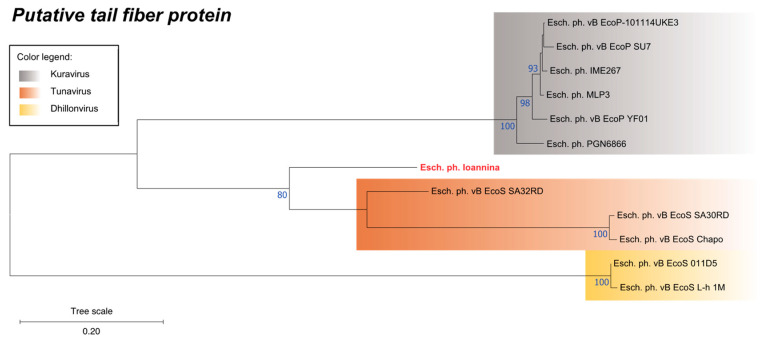
Phylogenetic tree of the Escherichia phage Ioannina putative tail fiber protein constructed using the Maximum Likelihood method of the MEGA11 software. The “Esch. ph. Ioannina” represents the Escherichia phage Ioannina putative tail fiber protein. Bootstrap values (blue-colored text) were obtained from 100 bootstrap replicates, and only those above 70 are displayed next to each node. The tree scale is displayed on the bottom left corner of the phylogenetic tree.

The analysis of coding sequences of the Escherichia phage Ioannina showed the presence of a CDS (CDS28) that was not present in most of the studied members of the *Dhillonvirus* genus (Table 2). Construction of a phylogenetic tree that encompassed homologous proteins (or predicted proteins), from related phage families, revealed a closer relationship of CDS28 with members of the *Kuravirus* or the *Tunavirus* (*Drexlerviridae*) genera, which was additionally visualized via MUSCLE v. 3.8 alignment (Figure 6, Supplementary file S2). It is intriguing that similar CDSs in the *Dhillonvirus* genus, where Escherichia phage Ioannina is predicted to belong, are more distantly related to CDS28 (Figure 6).

## 4. Discussion

The focus of the bacteriophage research has in the past decade been either on the isolation and characterization of bacteriophages against pathogenic and multidrug resistant bacterial strains, or on the analysis of phageome from human or environmental samples [37,38]. A definite host–virus relationship has been reported only for a minority of phage metagenomes [39]. Moreover, our knowledge on host–virus relationships involving commensal bacteria (important for mucosal homeostasis) is even more limited [40]. Importantly, phage therapy is a promising alternative for the combat against multidrug-resistant strains as our antibiotics arsenal is significantly losing its potential [41]. Phages for phage therapy have been isolated from the environment [42,43,44,45]. As the host range of phages varies, phage therapy requires the creation of phage cocktails for the prompt treatment of diseases, caused by MDR bacteria. Phage cocktails may also overcome the potential emergence of phage resistance during treatment [46]. The combination of antibiotics and phage therapy has been shown to be more effective in treating serious bacterial infections, than mono-phage therapy [47].

In this study, we isolated and characterized a lytic bacteriophage from biological wastewater treatment from the University Hospital of Ioannina, Epirus, Greece, using the strain *E. coli* ATCC 25922 as the host bacterium. The identified phage was lytic against *E. coli* ATCC 25922, as well as for a variety of *E. coli* isolates of clinical or environmental origin. Based on the morphological characteristics (e.g., long non-contractile tail and icosahedral head), the novel phage, namely Escherichia phage Ioannina, was classified into siphoviruses of *Caudoviricetes*. This classification was further refined by whole-genome sequencing of the isolated virus, and the virus was found to belong to the *Dhillonvirus* genus. As the similarity with the other members of *Dhillonvirus* genome is marginal according to ICTV demarcation criteria, there is a possibility that the novel virus belongs to a novel genus. Similar phages from hospital sewage have been isolated in the past, infecting *E. coli* pathogenic strains 40371 (genus *Cornellvirus*) and O18 (genus *Dhillonvirus*) [48,49]. Lytic bacteriophages infecting pathogenic *E. coli* O157:H7 (genus *Kuttervirus*) and 6 clinical *E. coli* isolates (genus *Tequatrovirus*), belonging to *Ackermannviridae* and myoviruses, respectively, have also been isolated from hospital wastewater [50,51].

Phages within the *Dhillonvirus* genus, infecting various *E. coli* strains, have been isolated from different sources of environmental, animal or human origin. Specifically, a previous report indicated that four different phages, which belong to the *Dhillonvirus* genus, were isolated from wastewater treatment plants against *E. coli* K-12 MG1655 [52]. Also, phages belonging to the *Dhillonvirus* genus have been isolated from fecal samples of healthy cattle [53], pig farm [54], slurry of birds’ feces [55] and fresh goat fecal samples [56]. Escherichia phage Gluttony_ev152 (the phylogenetically closest relative of Escherichia phage Ioannina) was isolated from feces of children (LR597646). Finally, a phage belonging to the *Dhillonvirus* genus has been isolated from a possibly contaminated culture of the *E. coli* BL21 (DE3) laboratory strain [57]. 

Temperature and pH are two important factors for phage viability. Escherichia phage Ioannina was tested at different thermal and pH values, in order to evaluate its lytic activity. Thermal stability tests showed that the Escherichia phage Ioannina was stable up to 40 °C with a gradual drop of activity between 40 and 55 °C and almost complete inactivation at 60 °C. In addition, it indicated a similar thermal stability between 37 and 55 °C compared to other phages of the member of siphoviruses [48,58]. Escherichia phage Ioannina viability testing, at different pH values, showed higher stability at alkaline pH (pH 10). On the other hand, the phage was sensitive to lower pH values (pH < 4). This finding was in agreement with earlier reports, identifying several members of siphoviruses resistant to alkaline pH while sensitive to acidic pH [59,60,61]. 

Comparative genomic analysis revealed that the Escherichia phage Ioannina indicated the highest nucleotide similarity with the “*Siphoviridae* sp. CtFRY1 metagenome” (BK032676.1). Phylogenetic analysis for three annotated proteins (portal protein, large terminase subunit protein and major capsid protein) showed that the CtFRY1 partial genome formed a distinct cluster within the *Dhillonvirus* genus. As Escherichia phage Ioannina presented similarity close to 70% (nucleotide identity of the full length genome) compared to other representatives of the *Dhillonvirus* genus, it is anticipated that the identification of more related phages, in the future, will lead into the emergence of a novel genus or subgenus [62]. 

Finally, in an attempt to identify the source of the similarity divergence between the Escherichia phage Ioannina and the other representatives from the *Dhillonvirus* genus, we characterized a gene (CDS28) absent in the vast majority of the species within the genus. CDS28 is predicted to encode a putative tail fiber protein. Tail fiber proteins are located at the tip of the tail and are responsible for the phage binding to a specific receptor present on the bacterial cell surface, such as lipopolysaccharides (LPS), teichoic acids and organelles [63]. Tail fiber proteins determine the host range during infection process [64]. Changes in tail fiber proteins have been shown to lead to a change in phage specificity towards the species of bacteria it infects [65].

Phylogenetic analysis of this protein, from CDS28, revealed that this gene showed a higher similarity with the *Kuravirus* genus of podoviruses (72.0–74.2%) and the *Tunavirus* genus of the family *Drexlerviridae* (47.0–72.5%). Interestingly, the similarity of this putative protein, with other related proteins in the *Dhillonvirus* genus, was only 37.8–38.7%. This observation may indicate a possible recombination event with either *Kuravirus* or *Tunavirus* (*Drexlerviridae*). Recombination in tail fiber protein genes have been observed much more frequently than in other regions of phage genomes, suggesting adaptive pressure towards switch of phage specificity as these proteins are involved in the interaction with the host cell [66]. In another study, representatives of *Litunavirus* genus (*Schitoviridae*) were analyzed, and the putative tail fiber region was shown to be a hotspot of recombinations with multiple phages species incorporating this region from other genera [67].

In conclusion, this study isolated and characterized a lytic bacteriophage against *E. coli* ATCC 25922 that is also efficiently lytic against a variety of *E. coli* isolates of clinical or environmental origin. Genomic analysis of the phage revealed that this novel phage, namely Escherichia phage Ioannina, belongs to a distant cluster of Dhillonviruses with a unique CDS encoding for a putative tail fiber protein. There is evidence that this CDS was incorporated through a recombination event from a different genus of phages. Escherichia phage Ioannina, due to its fast and potent lytic activity, may be used as a therapeutic tool against MDR *E. coli* strains, either alone or within a phage cocktail. Escherichia phage Ioannina, lacking genes encoding known virulence factors or providing antibiotic resistance, serves as a good candidate for further clinical research as use.

## Figures and Tables

**Table 1:** Lytic activity of the Escherichia phage Ioannina on different bacterial species (environmental, clinical and reference strains). The antibiotics resistance is reported as S (susceptible to antibiotics), R (resistance to one antimicrobial agent) and MDR (resistance to at least one antimicrobial agent in more than three categories). Phylogroups of *E. coli* isolates are reported as per Clermont’s schema. Phage lytic activity is presented as positive (+) or negative (−).

Sample No.	Bacterial Strains/ Isolates *	Samples Source	Antibiotics Resistance ^$^	Phylogroup ^&^	Phage Lytic Activity ^¶^
1	*Escherichia coli* 823	Wastewater treatment plant	S	A	+
2	*Escherichia coli* 668	Wastewater treatment plant	S	B1	+
3	*Escherichia coli* 824	Wastewater treatment plant	S	B2	−
4	*Escherichia coli* 663	Wastewater treatment plant	S	D	
5	*Escherichia coli* 792	Wastewater treatment plant	MDR	A	−
6	*Escherichia coli* 494	Wastewater treatment plant	MDR	B1	−
7	*Escherichia coli* 810	Wastewater treatment plant	MDR	B2	−
8	*Escherichia coli* 638	Wastewater treatment plant	MDR	D	−
9	*Escherichia coli* 640	Wastewater treatment plant	R	A	−
10	*Escherichia coli* 643	Wastewater treatment plant	R	B1	−
11	*Escherichia coli* 809	Wastewater treatment plant	R	B2	−
12	*Escherichia coli* 635	Wastewater treatment plant	R	D	−
13	*Escherichia coli* 865	Hospital wastewater	WT	A	+
14	*Escherichia coli* 866	Hospital wastewater	WT	B1	−
15	*Escherichia coli* 843	Hospital wastewater	WT	B2	−
16	*Escherichia coli* 580	Hospital wastewater	WT	D	−
17	*Escherichia coli* 426	Hospital wastewater	MDR	D	+
18	*Escherichia coli* 858	Hospital wastewater	MDR	A	−
19	*Escherichia coli* 546	Hospital wastewater	MDR	B2	−
20	*Escherichia coli* 576	Hospital wastewater	R	B2	−
21	*Escherichia coli* 545	Hospital wastewater	R	D	+
22	*Escherichia coli* 674	Hospital wastewater	R	A	−
23	*Escherichia coli* 759	River water	WT	A	−
24	*Escherichia coli* 774	River water	WT	B1	−
25	*Escherichia coli* 624	River water	WT	B2	−
26	*Escherichia coli* 769	River water	WT	D	−
27	*Escherichia coli* 472	River water	MDR	A	−
28	*Escherichia coli* 607	River water	MDR	B1	−
29	*Escherichia coli* 737	River water	MDR	B2	−
30	*Escherichia coli* 408	River water	MDR	D	−
31	*Escherichia coli* 614	River water	R	A	−
32	*Escherichia coli* 372	River water	R	D	−
33	*Escherichia coli* 743	River water	R	B2	−
34	*Escherichia coli* 784	River water	R	B1	−
35	*Escherichia coli* 117	Clinical	MDR	A	+
36	*Escherichia coli* 60	Clinical	MDR	B2	+
37	*Escherichia coli* 203	Clinical	MDR	D	+
38	*Escherichia coli* 325	Clinical	R	A	+
39	*Escherichia coli* 5	Clinical	R	A	−
40	*Escherichia coli* 264	Clinical	R	B1	−
41	*Escherichia coli* 294	Clinical	R	B2	−
42	*Escherichia coli* 378	Clinical	R	B2	+
43	*Escherichia coli* 313	Clinical	R	D	−
44	*Escherichia coli* 324	Clinical	S	A	+
45	*Escherichia coli* 368	Clinical	S	D	−
46	*Escherichia coli* 387	Clinical	S	B2	−
47	*Escherichia coli* 301	Clinical	S	B1	−
48	*Escherichia coli* 25922	Reference strain from Becton Dickinson, France S.A.S	S	−	+
49	*Escherichia coli* 35218	Reference strain from Becton Dickinson, France S.A.S	S	−	−
50	*Escherichia coli* 13846	Reference strain from Becton Dickinson, France S.A.S	S	−	−
51	*Escherichia coli* O157:H7	Clinical	MDR	−	−
52	*Klebsiella pneumoniae* 13883	Reference strain from Becton Dickinson, France S.A.S	S	−	−
53	*Klebsiella pneumoniae* 700603	Reference strain from Becton Dickinson, France S.A.S	S	−	−
54	*Pseudomonas aeruginosa* 27853	Reference strain from Becton Dickinson, France S.A.S	S	−	−
55	*Yersinia enterocolitica* 9610	Reference strain from Becton Dickinson, France S.A.S	S	−	−
56	*Acinetobacter baumannii* 17978	Reference strain from Becton Dickinson, France S.A.S	S	−	−
57	*Acinetobacter baumannii* 19668	Reference strain from Becton Dickinson, France S.A.S	S	−	−

* The number of the isolate is the respective number as reported in Dioli et al. https://pubmed.ncbi.nlm.nih.gov/37374900/ (accessed on 23 July 2024). ^$^ Antibiotics resistance is reported as S (susceptible to antibiotics), R (resistance to one antimicrobial agent) and MDR (resistance to at least one antimicrobial agent in more than three categories). Resistances have been previously reported, as reported in Dioli et al. https://pubmed.ncbi.nlm.nih.gov/37374900/ (accessed on 23 July 2024). **^&^** Phylogroups of *E. coli* isolates are reported as per Clermont’s schema, as identified in Dioli et al. https://pubmed.ncbi.nlm.nih.gov/37374900/ (accessed on 23 July 2024). ^¶^ Phage lytic activity is presented as positive (+) or negative (−).

**Table 2:** Closest putative tail fiber amino acid sequences to the Escherichia phage Ioannina putative tail fiber protein, based on the BLASTp tool.

Phage Name	BLASTp Coverage (%)	BLASTp Percent Identity (%)	Genus	Family/ Morphotype	NCBI Accession Number
**vB_EcoS_SA32RD**	98	72.5	*Tunavirus*	*Drexlerviridae*	UIU27553.1
**PGN6866**	92	74.22	*Kuravirus*	podoviruses	QKL16987.1
**vB_EcoP_YF01**	92	72.89	*Kuravirus*	podoviruses	WBF04932.1
**IME267**	92	72.44	*Kuravirus*	podoviruses	YP_010673185.1
**MLP3**	92	72.89	*Kuravirus*	podoviruses	UEN68517.1
**vB_EcoP-101114UKE3**	92	72.44	*Kuravirus*	podoviruses	YP_010673043.1
**νB_EcoP_SU7**	92	72	*Kuravirus*	podoviruses	YP_010672804.1
**vB_EcoS_011D5**	99	38.7	*Dhillonvirus*	siphoviruses	QMP82830.1
**vB_EcoS_L-h 1M**	99	37.79	*Dhillonvirus*	siphoviruses	UNY42316.1
**vB_EcoS_SA30RD**	72	48.02	*Tunavirus*	*Drexlerviridae*	UIU27628.1
**vB_EcoS_Chapo**	72	47.46	*Tunavirus*	*Drexlerviridae*	QLF82390.1

## Data Availability

The raw sequencing datasets for the current study are available in the NCBI Sequence Read Archive repository, under the Bioproject with accession number PRJNA941078 (NCBI BioProject database, https://www.ncbi.nlm.nih.gov/bioproject/PRJNA941078, last accessed date 13 March 2023). The assembled genome of the phage identified in the study is available in the NCBI Nucleotide repository and can be accessed online via the GenBank accession number OQ589852 (NCBI Nucleotide database, https://www.ncbi.nlm.nih.gov/nucleotide, last accessed date 13 March 2023). All custom scripts developed exclusively for the purposes of this study were uploaded to Github and can be accessed online (Github, https://github.com/konskons11/MOSQ, last accessed date 13 March 2023).

## Supplementary Materials

The following supporting information can be downloaded at: https://www.mdpi.com/article/10.3390/cimb46090551/s1. Figure S1. Phylogenetic tree of the Escherichia phage Ioannina terminase large subunit protein constructed using the Maximum-Likelihood method of the MEGA11 software. The “Esch. ph. Ioannina” represents the Escehrichia phage Ioannina terminase large subunit protein. Bootstrap values (blue coloured text) were obtained from 100 bootstrap replicates and only those above 70 are displayed next to each node. Tree scale is displayed on the bottom left corner of the phylogenetic tree. Figure S2. Phylogenetic tree of the Escherichia phage Ioannina major capsid protein constructed using the Maximum-Likelihood method of the MEGA11 software. The “Esch. ph. Ioannina” represents the Escherichia phage Ioannina major capsid protein. Bootstrap values (blue coloured text) were obtained from 100 bootstrap replicates and only those above 70 are displayed next to each node. Tree scale is displayed on the bottom left corner of the phylogenetic tree. Figure S3. Heatmap generated by VIRIDIC tool incorporating intergenomic similarity values (right half) and alignment indicators (left half and top annotation). In the right half, the color-coding allows a rapid visualization of the clustering of the phage genomes based on intergenomic similarity: the more closely-related the genomes, the darker the color. The numbers represent the similarity values for each genome pair, rounded to the first decimal. In the left half, three indicator values are represented for each genome pair, in the order from top to bottom: aligned fraction genome 1 (for the genome found in this row), genome length ratio (for the two genomes in this pair) and aligned fraction genome 2 (for the genome found in this column). The darker colors emphasize low values, indicating genome pairs where only a small fraction of the genome was aligned (orange to white color gradient), or where there is a high difference in the length of the two genomes (black to white color gradient). The aligned genome fractions are expected to decrease with increasing the distance between the phages. Therefore, darker colors should correspond to genome pairs with low similarity values, and whiter colors to genome pairs with higher similarity values. Similarly, more closely-related phages are expected to have similar lengths. A 95% threshold was used for the species level demarcation and a 70% threshold for the genera level. According to the heatmap, Escherichia phage Ioannina belongs to the Dhillonvirus genus. The genome of the Escherichia phage Ioannina and the genome of other related dhillonviruses have 72.1 to 77.9 similarity. Table S1. List of Escherichia phage Ioannina predicted CDSs, their positions on the phage genome, respective length, BLASTp annotation, putative role in phage life cycle and predicted protein size. Table S2. BLASTn alignment statistics of phage entries closely related to the Escherichia phage Ioannina identified in this study. Nucleotide identity full-length genome value represents the value of “Query coverage” (x) “Nucleotide identity” for each phage. Supplementary File S1. Genome organization synteny plot of Escherichia phage Ioannina compared to selected highly similar phages of the genus Dhillonvirus at the nucleotide level. Supplementary File S2. MUSCLE (v3.8) amino acid sequence alignment of the putative tail fiber protein encoded by the isolated Escherichia phage Ioannina against phylogenetically closely related Escherichia phages.
