# Supplementary material for: A Novel Dhillonvirus Phage against Escherichia coli Bearing a Unique Gene of Intergeneric Origin
Source: Curr Issues Mol Biol. 2024 Aug 23;46(9):9312–29. doi: 10.3390/cimb46090551 (PMC11430396; doi:10.3390/cimb46090551)
Supplement: Supplementary file 1 [file cimb-46-00551-s001.zip › Supplementary file S2.pdf]

Putative tail fiber protein | CLUSTAL multiple sequence alignment by MUSCLE (3.8)

Color legend:
- Dhillonvirus
- Kuravirus
- Tunavirus

```
Esch._ph._vB_EcoS_011D5          ---MAAGTLSVTNN---------------SKAVVGVGTTFTTFTA----GDFLTLVVGQV
Esch._ph._vB_EcoS_L-h_1M         ---MAAGTLSVTNN---------------SKAVVGVGTTFTTFTA----GDFLTLVVGQV
Esch._ph._vB_EcoS_SA30RD         MALYRRGTASMDAD---------------G-TVHGTDTKWKDQLALIRVGATIVFLEQPI
Esch._ph._vB_EcoS_Chapo          MALYRRGTASMDAD---------------G-TVHGTDTKWKDQLALIRVGATIVFLEQPI
Esch._ph._PGN6866                MIVYNNQAPDAVNNVGQFGATEGSIGAYKQAAEYAADSKYWALLAESKFGTIDDLIAEVE
Esch._ph._vB_EcoP_YF01           MIVYNNQAPDAVNNVGQFGATEGSIGAYKQAAEYAADSKYWALLAESKFGTIDDLIAEVE
Esch._ph._vB_EcoP_SU7            MIVYNNQAPDAVNNVGQFGATEGSIGAYKQAAEYAADSKYWALLAESKFGTIDDLIAEVE
Esch._ph._MLP3                   MIVYNNQAPDAVNNVGQFGATEGSIGAYKQAAEYAADSKYWALLAESKFGTIDDLIAEVE
Esch._ph._IME267                 MIVYNNQAPDAVNNVGQFGATEGSIGAYKQAAEYAADSKYWALLAESKFGTIDDLIAEVE
Esch._ph._vB_EcoP-101114UKE3     MIVYNNQAPDAVNNVGQFGATEGSIGAYKQAAEYAADSKYWALLAESKFGTIDDLIAEVE
Esch._ph._Ioannina               ------------------------------------------------------------
Esch._ph._vB_EcoS_SA32RD         MALYRRGTASMDAD---------------G-TVHGTDTKWKDQLALIRVGATIVFLEQPI

Esch._ph._vB_EcoS_011D5          PY----------------------------------------------------------
Esch._ph._vB_EcoS_L-h_1M         PY----------------------------------------------------------
Esch._ph._vB_EcoS_SA30RD         KL----------------------------------------------------------
Esch._ph._vB_EcoS_Chapo          KL----------------------------------------------------------
Esch._ph._PGN6866                RLYQQGVLMKQDIEDLKQDFKDQDARLMTLIAQTNAAVSDANNAVALINQKLIEVQNQLD
Esch._ph._vB_EcoP_YF01           RLYQQGVLMKQDIEDLKQDFIDQDARLMSLIAQTNAAVSDANNAVALINQKLIEVQKQLD
Esch._ph._vB_EcoP_SU7            RLYQQGVLMKQDIEDLKQDFKDQDARLMSLIAQTNAAVSDANNAVALINQKLIEVQNQLD
Esch._ph._MLP3                   RLYQQGVLMKQDIEDLKQDFKDQDARLMNLIAQTNAAVSDANNAVALINQKLIEVQKQLD
Esch._ph._IME267                 RLYQQGVLMKQDIEDLKQDFKDQDARLMSLIAQTNAAVSDANNAVALINQKLIEVQNQLD
Esch._ph._vB_EcoP-101114UKE3     RLYQQGVLMKQDIEDLKQDFKDQDARLMSLIAQTNAAVSDANNAVALINQKLIEVQNQLD
Esch._ph._Ioannina               ------------------------------------------------------------
Esch._ph._vB_EcoS_SA32RD         KL----------------------------------------------------------

Esch._ph._vB_EcoS_011D5          ------------------------------------------------------------
Esch._ph._vB_EcoS_L-h_1M         ------------------------------------------------------------
Esch._ph._vB_EcoS_SA30RD         ------------------------------------------------------------
Esch._ph._vB_EcoS_Chapo          ------------------------------------------------------------
Esch._ph._PGN6866                VLLGMSVDVTTLPPGTPATGSFNPNTGVISLGIPEGEPGKDGSVKDLDTAPTGVPELGDL
Esch._ph._vB_EcoP_YF01           VLLGMSVDVTTLPPGTPATGSFNPTTGVISLGIPEGEPGKDGSVKDLDTAPTGVPELGDL
Esch._ph._vB_EcoP_SU7            VLLGMSVDVTTLPPGTPATGSFNPNTGVISLGIPEGEPGKDGSVKDLDTAPTGVPELGDL
Esch._ph._MLP3                   VLLGMSVNVTTLPPGTPATGSFNPNTGVISLGIPEGEPGKDGSVKDLDTAPTGVPELGDL
Esch._ph._IME267                 VLLGMSVDVTTLPPGTPATGSFNPNTGVISLGIPEGDPGKDGSVKDLDTAPTGVPELGDL
Esch._ph._vB_EcoP-101114UKE3     VLLGMSVDVTTLPPGTPATGSFNPNTGVVSLGIPEGEPGKDGSVKDLDTAPTGVPELGDL
Esch._ph._Ioannina               ------------------------------------------------------------
Esch._ph._vB_EcoS_SA32RD         ------------------------------------------------------------

Esch._ph._vB_EcoS_011D5          ------------TVAIASVESDTAITLVLPFDGPTATGLAWDGVKRDTMSLATMGVTVQA
Esch._ph._vB_EcoS_L-h_1M         ------------TVAIASVESDTAITLVLPFDGPTATGLAWDGVKRDTMSLATMGVTVQA
Esch._ph._vB_EcoS_SA30RD         ------------AVISDIVSDT--ELKAISTDGQTASDGKYVILLNDSLTVNGLAQNVAE
Esch._ph._vB_EcoS_Chapo          ------------AVISDIVSDT--ELKAISTDGQTASDGKYVILLNDSLTVNGLAQNVAE
Esch._ph._PGN6866                GFYVDKDDNTVHKTTLENIANLIPSVRSVSVNGGPALDGEVALTINKETVGLGNVLNVAQ
Esch._ph._vB_EcoP_YF01           GFYVDKDDNTVHKTTLENIANLIPSVRSVSVNGGPALDGEVALTINKETVGLGNVLNVAQ
Esch._ph._vB_EcoP_SU7            GFYVDKDDNTVHKTTLENIANLIPSVRSVSVNGGPALDGEVALTINKETVGLGNVLNVAQ
Esch._ph._MLP3                   GFYVDKDDNTVHKTTLENIANLIPSVRSVSVNGGPALDGEVALTLNKETVGLGNVLNVAQ
Esch._ph._IME267                 GFYVDKDDNTVHKTTLDNIANLIPSVRSVSINGGPALDGEVALTVNKETVGLGNVLNVAQ
Esch._ph._vB_EcoP-101114UKE3     GFYVDKDDNTVHKTTLENIANLIPSVRSVSVNGGPALDGEVALTINKETVGLGNVLNVAQ
Esch._ph._Ioannina               ------------------------------------------------------------
Esch._ph._vB_EcoS_SA32RD         ------------AVISDIVSDT--ELKAISTDGQTAADGKYVILLNDSLTVNGLAQNVAE

Esch._ph._vB_EcoS_011D5          ----------QKALRLMIADENN-------------------------------------
Esch._ph._vB_EcoS_L-h_1M         ----------QKALRLMIADENN-------------------------------------
Esch._ph._vB_EcoS_SA30RD         ------------TLRYYQSKETE-------------------------------------
Esch._ph._vB_EcoS_Chapo          ------------TLRYYQSKETE-------------------------------------
Esch._ph._PGN6866                YSRQEINDKFDKTTKTYQSKAEADADAQYRQVGEKVLVWEATKYEFYTVAANKTLTPVKT
Esch._ph._vB_EcoP_YF01           YSRQEINDKFDKTTKTYQSKAEADADAQYRQVGEKVLVWEATKYEFYTVAANKTLTPVKT
Esch._ph._vB_EcoP_SU7            YSRQEINDKFDKTTKTYQSKAEADADAQYRQVGEKVLVWEATKYEFYTVAANKTLTPVKT
Esch._ph._MLP3                   YSRQEINDKFDKTTKTYQSKAEADADAQYRQVGEKVLVWEATKYEFYTVAANKTLTPVKT
Esch._ph._IME267                 YSRQEINDKFDKTTKTYQSKAEADADAQYRQVGEKVLVWEATKYEFYTVAANKTLTPVKT
Esch._ph._vB_EcoP-101114UKE3     YSRQEINDKFDKTTKTYQSKAEADADAQYRQVGEKVLVWEATKYEFYTVAANKTLTPVKT
Esch._ph._Ioannina               ------------------------------------------------------------
Esch._ph._vB_EcoS_SA32RD         ------------TLRYYQSRETE-------------------------------------

Esch._ph._vB_EcoS_011D5          ------------------------------------------------------------
Esch._ph._vB_EcoS_L-h_1M         ------------------------------------------------------------
Esch._ph._vB_EcoS_SA30RD         ------------------------------------------------------------
Esch._ph._vB_EcoS_Chapo          ------------------------------------------------------------
Esch._ph._PGN6866                EGRILTVNSRSPDSSGNIDITIPTGNPSLYLGEMVMFPYDPTKNISYPGVLPADGRLVSK
Esch._ph._vB_EcoP_YF01           EGRILTVNSRSPDSSGNIDITIPTGNPSLYLGEMVMFPYDPTKNISYPGVLPADGRLVSK
Esch._ph._vB_EcoP_SU7            EGRILTVNSRSPDSSGNIDITIPTGNPSLYLGEMVMFPYDPSKNISYPGVLPADGRLVSK
Esch._ph._MLP3                   EGRILTVNSRSPDSSGNIDITIPTGNPSLYLGEMVMFPYDPTKNISYPGVLPADGRLVSK
Esch._ph._IME267                 EGRILTVNSRSPDSSGNIDITIPTGNPSLYLGEMVMFPYDPTKNISYPGVLPADGRLVSK
Esch._ph._vB_EcoP-101114UKE3     EGRILTVNSRSPDSSGNIDITIPTGNPSLYLGEMVMFPYDPSKNISYPGVLPADGRLVSK
Esch._ph._Ioannina               ------------------------------------------------------------
Esch._ph._vB_EcoS_SA32RD         ------------------------------------------------------------
```

```
Esch._ph._vB_EcoS_011D5       -------WRAIFGDEEEVTVTLPNGQVMQGMSWGYLSQLMNQIDPVEMRNLQQQAAASEA
Esch._ph._vB_EcoS_L-h_1M      -------WRAIFGDEEEVTVTLPNGQVMQGMSWGYLSQLMNQIDPVEMRNLQQQAAASEA
Esch._ph._vB_EcoS_SA30RD      ----------IAGAMDIIASLDMD-------NLNRIVEEIKA-NKSAAESAQNQAELARD
Esch._ph._vB_EcoS_Chapo       ----------IAGAMDIIASLDMD-------NLNRIVEEIKA-NKSAAESAQNQAELARD
Esch._ph._PGN6866             ESASDLGPSLVSGQLPVVSETEWQAGAKQYFSWGKLADGITDADSTNFINIRLPDWTGGE
Esch._ph._vB_EcoP_YF01        ESASDLGPSLVSGQLPVVSETEWQAGAKQYFSWGKLADGITDADSTNFINIRLPDWTGGE
Esch._ph._vB_EcoP_SU7         ESASDLGPSLVSGQLPVVSETEWQSGAKQYFSWGKLADGITDADSTNFINIRLPDWTGGE
Esch._ph._MLP3                ESASDLGPSLVSGQLPVVSETEWQAGAKQYFSWGKLADGITDADSTNFINIRLPDWTGGE
Esch._ph._IME267              ESASDLGPSLVSGQLPVVSETEWQAGAKQYFSWGKLADGITDADSTNFINIRLPDWTGGE
Esch._ph._vB_EcoP-101114UKE3  ESASDLGPSLVSGQLPVVSETEWQSGAKQYFSWGKLADGITDADSTNFINIRLPDWTGGE
Esch._ph._Ioannina            ------------------------------------------------------------
Esch._ph._vB_EcoS_SA32RD      ----------IAEAVELIKNLDMD-------KLNQIVADVNQ-AKSDAQDAQSQAELARD

Esch._ph._vB_EcoS_011D5       AAQGFRNEAEGFKTETAGIRDA---------TNQIKADTQAIHDATNTIKTQTAQIKTDT
Esch._ph._vB_EcoS_L-h_1M      AAQGFRNEAEGFKTETAGIRDA---------TNQIKADTQAIHDATNTIKTQTAQIKTDT
Esch._ph._vB_EcoS_SA30RD      SANAARDEAKATKGQVQQIVDGAVGSINAAKEQAITDVGSKESAAITHIDTEEKAAIKAI
Esch._ph._vB_EcoS_Chapo       SANAARDEAKATKGQVQQIVDGAVGSINAAKEQAITDVGSKESAAITHIDTEEKAAIKAI
Esch._ph._PGN6866             AIRAPDSDKDSQYNGSVQAQKPYVVTVN---NQAPDEITGNVNISRSILGAASSGANSDI
Esch._ph._vB_EcoP_YF01        AIRAPDSDKDSQYNGSVQAQKPYVVTVN---NQAPDEITGNVNISRSILGAAASGTNSDI
Esch._ph._vB_EcoP_SU7         AIRAPDSDKDSQYNGSVQAQKPYVVTVN---NQAPDEITGNVNISRSILGAASSGANSDI
Esch._ph._MLP3                AIRAPDSDKDSQYNGSVQAQKPYVVTVN---NQAPDEITGNVNISRSILGAASSGANSDI
Esch._ph._IME267              AIRAPDSDKDSQYNGSVQAQKPYVVTVN---NQAPDEITGNVNISRSILGAASSGANSDI
Esch._ph._vB_EcoP-101114UKE3  AIRAPDSDKDSQYNGSVQAQKPYVVTVN---NQAPDEITGNVNISRSILGAASSGANSDI
Esch._ph._Ioannina            ------------------------------------------------------------
Esch._ph._vB_EcoS_SA32RD      AANAARDETNSIKDQTQQIVDGAVDGINAAKDQAIADVGDKESAAVTHIDTEEAAAIQAI

Esch._ph._vB_EcoS_011D5       QAIKGQTDQI-KTDTGVIRDEANAAKAEAQAASTAALGY---------------------
Esch._ph._vB_EcoS_L-h_1M      QAIKGQTDQI-KTDTGVIRDEANAGKAEAQAASTAALGY---------------------
Esch._ph._vB_EcoS_SA30RD      NDAKGDLSGY-VNNAQAAAQTATSAKNDAQAARDDAVSA---------------------
Esch._ph._vB_EcoS_Chapo       NDAKGDLSGY-VNNAQAAAQTATSAKNDAQAARDDAVSA---------------------
Esch._ph._PGN6866             TSLTGLTTALSIAQGGTGGKTPSEARANLNLERFQQDNSQTLIYSPDYARRVYVDNTGGS
Esch._ph._vB_EcoP_YF01        TSLTGLTTALSVTQGGTGAKDAASARSNLGLSSIATLNTIPIANGGTGATTIDAARSNLS
Esch._ph._vB_EcoP_SU7         TSLSGLTTPLSISQGGTGAKNAASARSNLGLGSTATLNTIPVANGGTGATTVDVARSNLS
Esch._ph._MLP3                TSLSGLTTPLSISQGGTGAKDAAGARSNLGLGSTATLNTIPVANGGTGATTVDVARSNLS
Esch._ph._IME267              TSLSGLTTPLSISQGGTGAKDAAGARSNLGLGSIATLNTIPVANGGTGANTVDVARSNLS
Esch._ph._vB_EcoP-101114UKE3  TSLSGLTTPLSISQGGTGAKDAAGARSNLGLGSTATLNTIPVANGGTGATTVDVARSNLS
Esch._ph._Ioannina            ------------------------------------------------------------
Esch._ph._vB_EcoS_SA32RD      NDAKGDLSGY-VTDAQAAAQTATSAKNDAQAARDASVSA---------------------

Esch._ph._vB_EcoS_011D5       -----------------------------------RN------------EAEQFKNE---
Esch._ph._vB_EcoS_L-h_1M      -----------------------------------RN------------EAEQFKNE---
Esch._ph._vB_EcoS_SA30RD      ----------------------------KDAASVSAQ------------EAKDAANS---
Esch._ph._vB_EcoS_Chapo       ----------------------------KDAASVSAQ------------EAKDAANS---
Esch._ph._PGN6866             ----------------------------WGCQNVTDGGFIALGIPQGGTGAKDAAGARSN
Esch._ph._vB_EcoP_YF01        IDRVDQASGESRLLSPNKETYLFVDNNGWGCYSISPG------------RVGDLALG---
Esch._ph._vB_EcoP_SU7         IDRIDQASGESKLLSPNKETYLFVDNNGWGCYSTSAG------------RVGDVALS---
Esch._ph._MLP3                IDRIDQASGESKLLSPNKETYLFVDNNGWGCYSTSAG------------RVGDVALS---
Esch._ph._IME267              IDRIDQASGESKLLSPNKETYLFVDNNGWGCYSTSAG------------RVGDVALS---
Esch._ph._vB_EcoP-101114UKE3  IDRIDQASGESKLLSPNKETYLFVDNNGWGCYSTSAG------------RVGDVALS---
Esch._ph._Ioannina            ------------------------------------------------------------
Esch._ph._vB_EcoS_SA32RD      ----------------------------KDAAAVSAQ------------EAKDAAGS---

Esch._ph._vB_EcoS_011D5       -------------IDPSNFLAKG----------GNLAGLTDLPESR------------DN
Esch._ph._vB_EcoS_L-h_1M      -------------IDPSNFLAKG----------GNLAGLTDLPESR------------DN
Esch._ph._vB_EcoS_SA30RD      -------------INADNLLTKD----------GNLSGLTNKSVAR-----TNLDIDKFG
Esch._ph._vB_EcoS_Chapo       -------------INADNLLTKD----------GNLSGLTNKSVAR-----TNLDIDKFG
Esch._ph._PGN6866             LGLGSVSTLNNIPVANGGTGATTAAGARSNLGLGSVSTLDNVPIASGGTGAGDAAGARFN
Esch._ph._vB_EcoP_YF01        -------------VERGGTGAKDAASARSNLGLGSVSTLDNVPIASGGTGAGDAAGARFN
Esch._ph._vB_EcoP_SU7         -------------VERGGTGAKDAAGARSNLGLGSVSTLDNVPIASGGTGAGDAAGARFN
Esch._ph._MLP3                -------------VERGGTGAKDAAGARSNLGLGSVSTLDNVPIASGGTGAGDAAGARFN
Esch._ph._IME267              -------------VERGGTGAKNAASARSNLGLGSVSTLDNVPIASGGTGAGDAAGARFN
Esch._ph._vB_EcoP-101114UKE3  -------------VERGGTGAKNAASARSNLGLGSVSTLDNVPIASGGTGAGDAAGARFN
Esch._ph._Ioannina            -------------------MDKS----------ANLSDLADKTEAR------------SH
Esch._ph._vB_EcoS_SA32RD      -------------INADNLLTKS----------GNLDGITDAGEAR------------GH
                                                   .           ..:  : :   :

Esch._ph._vB_EcoS_011D5       LGLGALATK-------------DSAALNSTDTTG-ILPLDK-------------------
Esch._ph._vB_EcoS_L-h_1M      LGLGALATK-------------DSAALNSTDTTG-ILPIDK-------------------
Esch._ph._vB_EcoS_SA30RD      LGLQSSAGLVNSLSEGNSVNLANGFYVGNGDNFGKPFGIGDGQFLGVVTFNCTQGNNYKF
Esch._ph._vB_EcoS_Chapo       LGLQSSAGLVNSLSEGNSVNLANGFYVGEGGNFGKPFGIGDGQFLGVVTFNCTKGNNYKF
Esch._ph._PGN6866             LGLGSSATL------------------NTGTTSGNVLKVGD-------------------
Esch._ph._vB_EcoP_YF01        LGLGSSATL------------------NTGTTSGNVLKVGD-------------------
Esch._ph._vB_EcoP_SU7         LGLGNSATM------------------NTGTNSDNVLKVGD-------------------
Esch._ph._MLP3                LGLGNSATM------------------NTGTNSDNVLKVGD-------------------
Esch._ph._IME267              LGLGNSATM------------------NTGTNSDNVLKVGD-------------------
Esch._ph._vB_EcoP-101114UKE3  LGLGNSATM------------------NTGTNSDNVLKVGD-------------------
Esch._ph._Ioannina            LGLGSAATL------------------DVGDSSGNAMKVGD-------------------
Esch._ph._vB_EcoS_SA32RD      LGLGSAATL------------------NAGSATGDVMRVGD-------------------
                              ***   *                    .     .  : :..
```

Color legend:

Dhillonvirus

Kuravirus

Tunavirus

```
Esch._ph._vB_EcoS_011D5        -------------------------------------GGLGAGTIPGAR-----------
Esch._ph._vB_EcoS_L-h_1M       -------------------------------------RGLGAGTIPGAR-----------
Esch._ph._vB_EcoS_SA30RD       QLSGSYSSSGRMMFRCMNNGNYGAWKEVLTSASPSVEYGITIKRSDQGRPNQSGYISVGG
Esch._ph._vB_EcoS_Chapo        QLSGSYSSSGRMMFRCMNNGNYGAWKEVLTSASPSVEYGITIKRSDQGRPNQSGYIGVGG
Esch._ph._PGN6866              -------------------------------------FGI-------GRP----------
Esch._ph._vB_EcoP_YF01         -------------------------------------FGI-------GRP----------
Esch._ph._vB_EcoP_SU7          -------------------------------------FGI-------GRP----------
Esch._ph._MLP3                 -------------------------------------FGI-------GRP----------
Esch._ph._IME267               -------------------------------------FGI-------GRP----------
Esch._ph._vB_EcoP-101114UKE3   -------------------------------------FGI-------GRP----------
Esch._ph._Ioannina             -------------------------------------FGL-------GRP----------
Esch._ph._vB_EcoS_SA32RD       -------------------------------------FGL-------GKP----------
                                                                      *:       ..

Esch._ph._vB_EcoS_011D5        ------------------------ETMHIYGHDATFGTINN-------ILTYFNNIAGNG
Esch._ph._vB_EcoS_L-h_1M       ------------------------ETMHIYGHDATFGTINN-------ILTYFNNIAGNG
Esch._ph._vB_EcoS_SA30RD       NDVHFKNGTSNRALQLRDDSSVAYDGRVIIDSDKTMVIGAGKDINQPNIGSWIKGMRDTG
Esch._ph._vB_EcoS_Chapo        NDVYFKNGTSNRALQLRDDSSVAYDGRVIIDSDKTMVIGAGKDINQPNIGAWIKGMRDTG
Esch._ph._PGN6866              ------------------------DGALVFDTTSQDQLLAG---------------LDTY
Esch._ph._vB_EcoP_YF01         ------------------------DGALVFDTTSQDQLLAG---------------LDTY
Esch._ph._vB_EcoP_SU7          ------------------------DGALVFDTTSQDQLLAG---------------LDTY
Esch._ph._MLP3                 ------------------------DGALVFDTTSQDQLLAG---------------LDTY
Esch._ph._IME267               ------------------------DGALVFDTTSQDQLLAG---------------LDTY
Esch._ph._vB_EcoP-101114UKE3   ------------------------DGALVFDTTSQDQLLAG---------------LDTY
Esch._ph._Ioannina             ------------------------NGAKVFYTTSQDTLLAG---------------LDQY
Esch._ph._vB_EcoS_SA32RD       ------------------------DGAYVFNTTSQDVLLAG---------------LDTN
                                                       :   :           .                .

Esch._ph._vB_EcoS_011D5        GLFSFRDGAVDAT--NTYKFGSAILFRTADTYGLLSVDH-NSGNVRASGGTVSGGIAATA
Esch._ph._vB_EcoS_L-h_1M       RLFSFRDGAVDAT--NTYKFGSAILFRTADTYGLLSVDH-NSGNVRASGGTVSGGIAATA
Esch._ph._vB_EcoS_SA30RD       GSRVMRNTGAASAGGANFQYNAAIYMLAGDTFGLLSVDYRNANVYSI--GANNSDLNNGV
Esch._ph._vB_EcoS_Chapo        GSRVMRNTGAASAGGANFQHNAAIYMLAGDTFGLLSVDYRNANVYSI--GANNSGLNSGV
Esch._ph._PGN6866              GLCVFRNNQQIAAPWDIWNYSSNLFFRAGDTYSMISIPFESAGKIKVFGGAAGSGWKTS-
Esch._ph._vB_EcoP_YF01         GLCVFRNNQQIAAPWDIWNYSSNLFFRAGDTYSMISIPFESAGKIKVFGGAEGSGWKTS-
Esch._ph._vB_EcoP_SU7          GLCVFRNNQQIAAPWDIWNYSSNLFFRAGDTYSMISIPFESAGKIKVFGGASGSGWKTS-
Esch._ph._MLP3                 GLCVFRNNQQIAAPWDIWNYSSNLFFRAGDTYSMISIPFESAGKIKVFGGAAGSGWKTS-
Esch._ph._IME267               GLCVFRNNQQIAAPWDIWNYSSNLFFRAGDTYSMISIPFESAGKIKVFGGASGSGWKTS-
Esch._ph._vB_EcoP-101114UKE3   GLCVFRNNQQIAAPWDIWNYSSNLFFRAGDTYSMISIPFESAGKIKVFGGASGSGWKTS-
Esch._ph._Ioannina             GLCVFRNDLQIAAPWDIWNYSSNLFFRAGDTYSMLSIPFEAAGKVKVFGGAAGYGWKTG-
Esch._ph._vB_EcoS_SA32RD       GLCVFRNNQQIAAPWDIWNYSSNLFFRAGDTYSMISIPFEQAGKIKVFGGVAGYGWKTS-
                                   :*:    ::    ::..: : : :.**:.::*: .  :.      *. . .

Esch._ph._vB_EcoS_011D5        Y-INNLWGTRNTTVDGNGFIKTASPIVKIFSDGAAETNDEAEGVTVTRISAGVYVVEGCL
Esch._ph._vB_EcoS_L-h_1M       Y-INNLWGTRNTTVDGNGFIKTASPIVKIFSDGAAETNDEAEGVTLTRISAGVYVVEGCL
Esch._ph._vB_EcoS_SA30RD       VQVNALWGTKNTTVDVNGFIKRASPIVRVKRD-SFETNDESEGCTVQRIGVGEYLIEGCT
Esch._ph._vB_EcoS_Chapo        VQVNALWGTKNTTVDGNGFIKRASPIVRVKRD-SFETNDESEGCTVQRIGVGEYLIEGCT
Esch._ph._PGN6866              ---RTVYDTVNTTVDVNGFIKAASPIVKVFHDGSFETNEQSDGVSVKKISTGVYLISGCL
Esch._ph._vB_EcoP_YF01         ---RTIYDTVNTTVDVNGFIKAASPIVKVFHDGSFETNEQSDGVSVKKVSTGVYLILGCL
Esch._ph._vB_EcoP_SU7          ---RTVYDTVNTTVDVNGFIKAASPIVKVFHDGSFETNEQSDGVSVKKISTGVYLISGCL
Esch._ph._MLP3                 ---RTVYDTVNTTVDVNGFIKAASPIVKVFHDGSFETNEQSDGVSVKKISTGVYLISGCL
Esch._ph._IME267               ---RTVYDTVNTTVDVNGFIKAASPIVKVFHDGSFETNEQSDGVSVKKISTGVYLISGCL
Esch._ph._vB_EcoP-101114UKE3   ---RTVYDTVNTTVDVNGFIKAASPIVKVFHDGSFETNEQSDGVSVKKISTGVYLISGCL
Esch._ph._Ioannina             ---RTLLDNTTTTIDTNGFVKQASPIVKVFGDGKFEVNEESKGASVDRISVGVYRISGVL
Esch._ph._vB_EcoS_SA32RD       ---RTIYDTVNTTVDPNGFIKTASPIVKVFKDGRFETNEQSEGAKVERISTGVYKVSGVL
                                  . : .. .**:* ***:* *****.:  *   *.*:::.* .: .:..* * : *

Esch._ph._vB_EcoS_011D5        GLNSDPSWGGPDGGIEIPTDVNKQPRLWVDYKVRADGSVEVRTYHRTHPHAPAFARNEIE
Esch._ph._vB_EcoS_L-h_1M       GLNSDPSWGGPDGGIEIPTDVNKQPRLWVDYKVRADGSVEVRTYHRTHPHAPAFARNEIE
Esch._ph._vB_EcoS_SA30RD       GLNSDLAWGGISGGIEIPVDINKQPLLWVDYEVNADGSIILKTFHRTHESAPKFARNDKE
Esch._ph._vB_EcoS_Chapo        GLNSDLAWGGISGGIEIPVDINKQPLLWVDYEVNADGSIILKTFHRTHESAPKFARNDKE
Esch._ph._PGN6866              GLNSDAGWGGVDGGFEIPIDRNKQPRVWLDYEVKEDGSLLIKTYHRTHSTSPAFARNELE
Esch._ph._vB_EcoP_YF01         GLNSDAGWGGVDGGFEIPIDRNKQPRVWLDYEVKEDGSLLIKTYHRTHSTSPAFARNELE
Esch._ph._vB_EcoP_SU7          GLNSDAGWGGVDGGFEIPIDRNKQPRVWLDYEIKEDGSILVKTYHRIHPTSPVFARNEIE
Esch._ph._MLP3                 GLNSDAGWGGVDGGFEIPIDRNKQPRVWLDYEVKEDGSLLIKTYHRTHSTSPAFARNELE
Esch._ph._IME267               GLNSDAGWGGVDGGFEIPIDRNKQPRVWLDYEVKEDGSLLIKTYHRTHSTSPAFARNELE
Esch._ph._vB_EcoP-101114UKE3   GLNSDAGWGGVDGGFEIPIDRNKQPRVWLDYEVKEDGSLLIKTYHRTHSTSPAFARNELE
Esch._ph._Ioannina             GLNSDAAWGGVGGGFEIPVDINKQPRVWVDFEVEPEGSIILKTYHRTHDTAPSFAKT---
Esch._ph._vB_EcoS_SA32RD       GLNSEALWGGVNCGFEIPVDVNKQPRIWLDYEVEHDGSIVVKTYHRTHYSSPSFASNKIE
                               ****:  *** . *:*** * **** :*:*:::  :**: :.*:** *  :* ** .

Esch._ph._vB_EcoS_011D5        GVAEGDPVDIPRGTFISVRVNMPE-----------------------------------
Esch._ph._vB_EcoS_L-h_1M       GVAEGDPVDIPRGTFISVRVNMPE-----------------------------------
Esch._ph._vB_EcoS_SA30RD       GYSNGDPIDIPNDTFVSIRVEMPPDSIYNTELERMEREVRNVAKRSEWEEAHATGCKEE
Esch._ph._vB_EcoS_Chapo        GYSNGDPIDIPNDVFVSIRVEMPPNSIYNTELERMEREVRNGAKRSEWEEAHATGCKEE
Esch._ph._PGN6866              GFSDGDPVDIPKDAFISVRVEMPSK----------------------------------
Esch._ph._vB_EcoP_YF01         GFSDGDPVDIPKDAFISVRVEMPSK----------------------------------
Esch._ph._vB_EcoP_SU7          GFSDGDPIDIPSDAFISVRVEMPSK----------------------------------
Esch._ph._MLP3                 GFSDGDPVDIPKDAFISVRVEMPSK----------------------------------
Esch._ph._IME267               GFSDGDPVDIPKDAFISVRVEMPSK----------------------------------
Esch._ph._vB_EcoP-101114UKE3   GFSDGDPVDIPKDAFISVRVEMPSK----------------------------------
Esch._ph._Ioannina             -----------------------------------------------------------
Esch._ph._vB_EcoS_SA32RD       GFSNGDPIDIPSDAFVSIRVNMP------------------------------------
```

Color legend:

- Dhillonvirus
- Kuravirus
- Tunavirus
