# Supplementary material for: A Novel Dhillonvirus Phage against Escherichia coli Bearing a Unique Gene of Intergeneric Origin
Source: Curr Issues Mol Biol. 2024 Aug 23;46(9):9312–29. doi: 10.3390/cimb46090551 (PMC11430396; doi:10.3390/cimb46090551)
Supplement: Supplementary file 1 [file cimb-46-00551-s001.zip › Supplementary.pdf]

SUPPLEMENTARY FIGURES

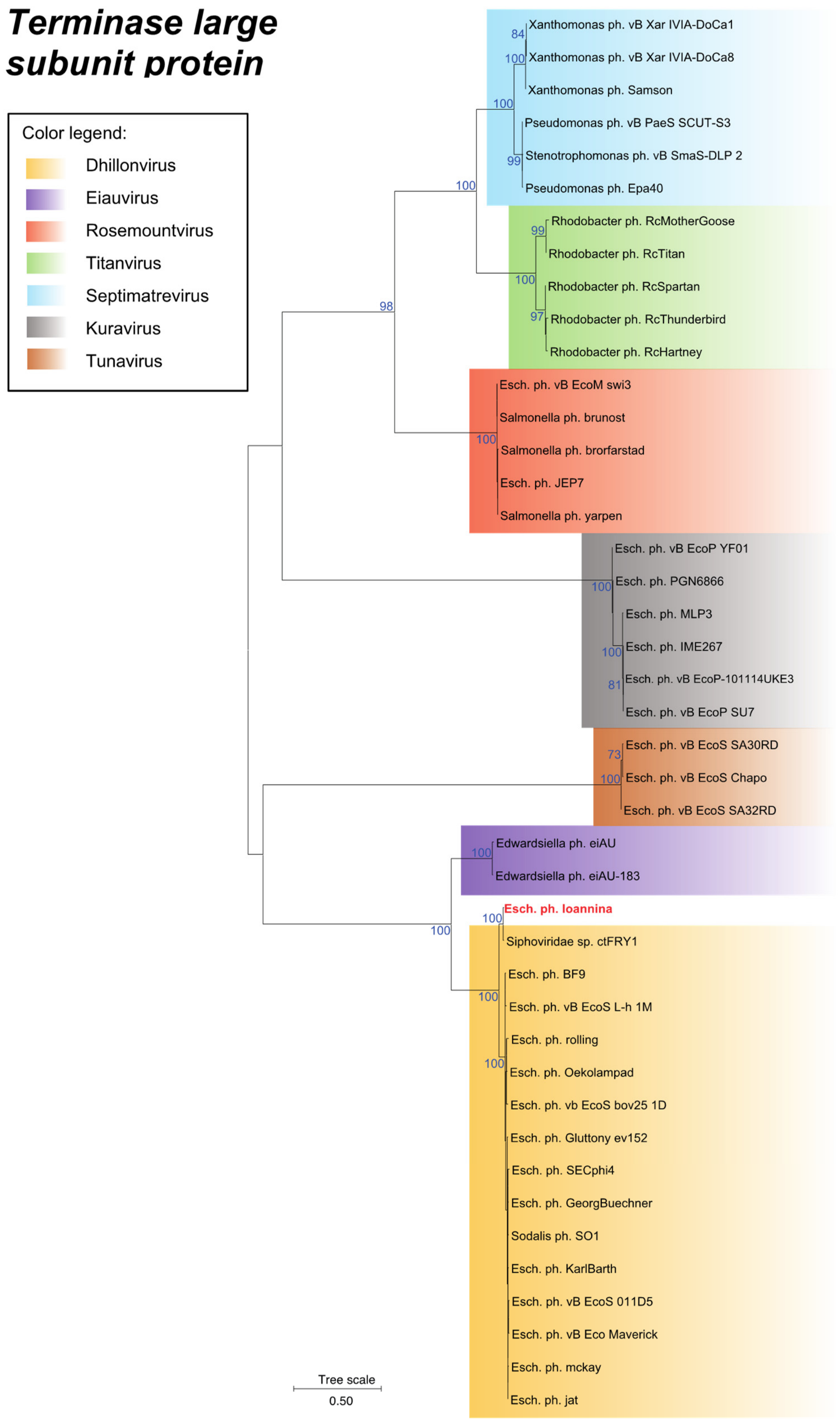

Figure S1. Phylogenetic tree of the Escherichia phage Ioannina terminase large subunit protein constructed using the Maximum-Likelihood method of the MEGA11 software. The "Esch. ph. Ioannina" represents the Escehrichia phage Ioannina terminase large subunit protein. Bootstrap values (blue coloured text) were obtained from 100 bootstrap replicates and only those above 70 are displayed next to each node. Tree scale is displayed on the bottom left corner of the phylogenetic tree.

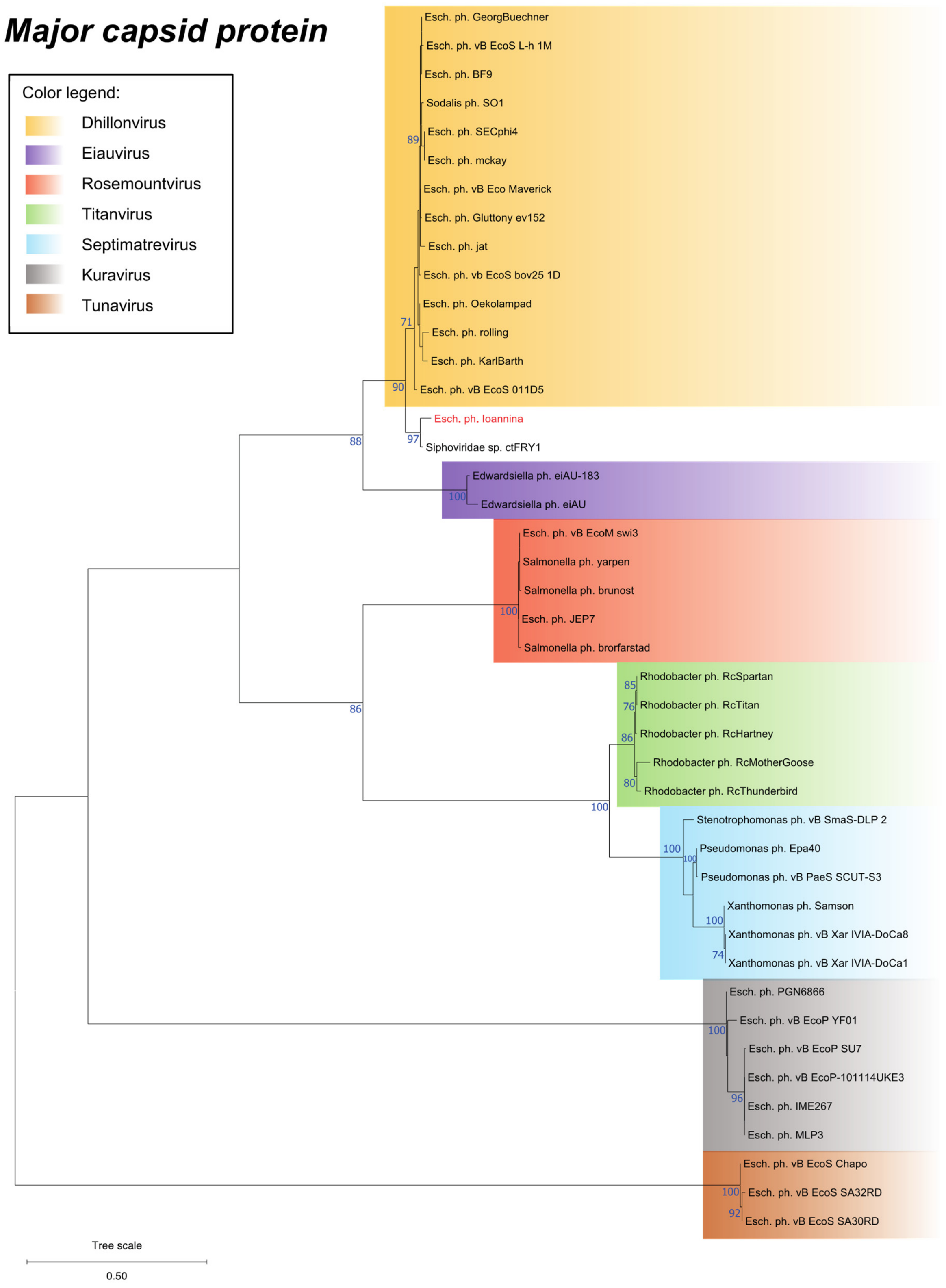

Figure S2. Phylogenetic tree of the Escherichia phage Ioannina major capsid protein constructed using the Maximum-Likelihood method of the MEGA11 software. The "Esch. ph. Ioannina" represents the Escherichia phage Ioannina major capsid protein. Bootstrap values (blue coloured text) were obtained from 100 bootstrap replicates and only those above 70 are displayed next to each node. Tree scale is displayed on the bottom left corner of the phylogenetic tree.

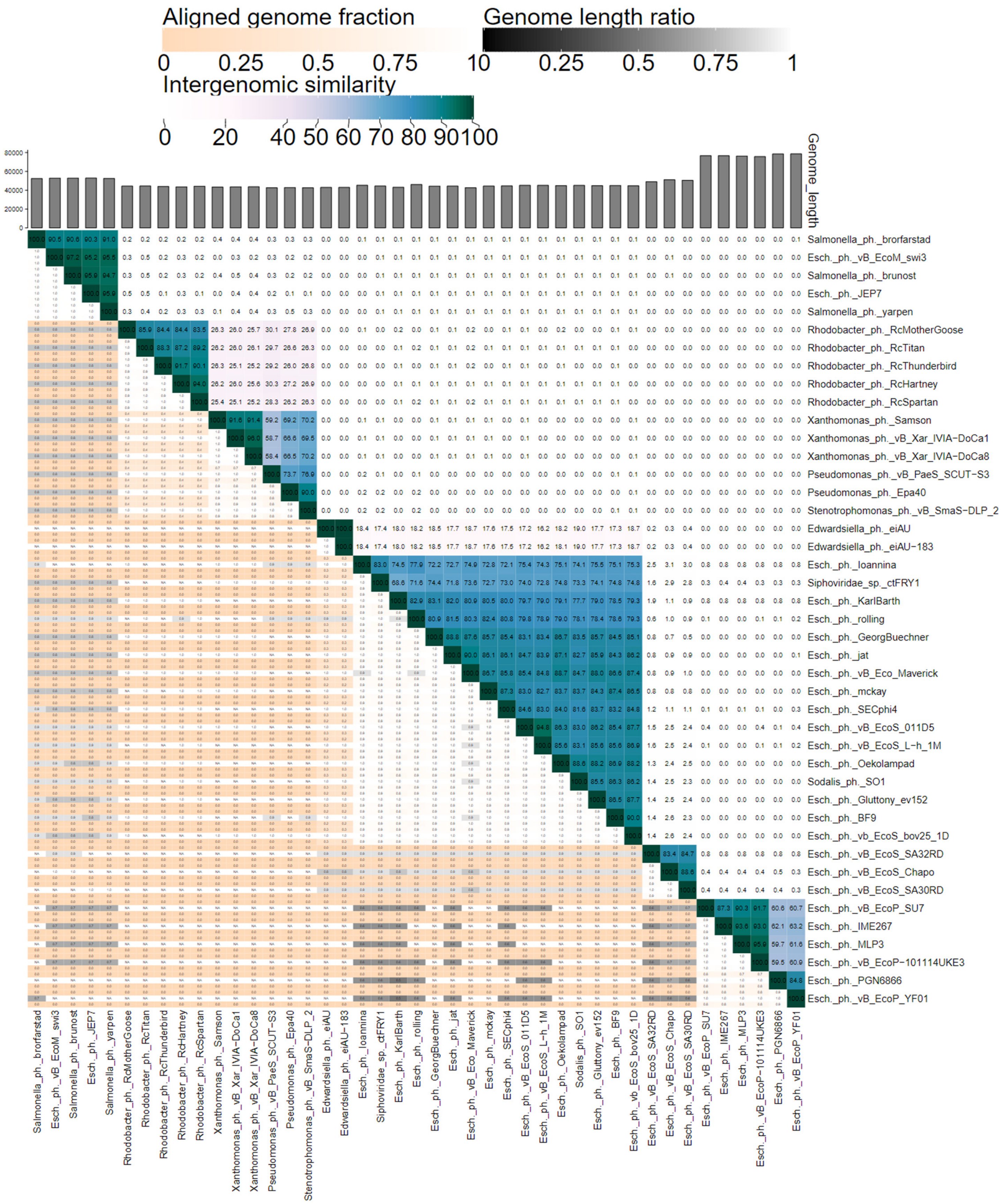

Figure S3. Heatmap generated by VIRIDIC tool incorporating intergenomic similarity values (right half) and alignment indicators (left half and top annotation). In the right half, the color-coding allows a rapid visualization of the clustering of the phage genomes based on intergenomic similarity: the more closely-related the genomes, the darker the color. The numbers represent the similarity values for each genome pair, rounded to the first decimal. In the left half, three indicator values are represented for each genome pair, in the order from top to bottom: aligned fraction genome 1 (for the genome found in this row), genome length

ratio (for the two genomes in this pair) and aligned fraction genome 2 (for the genome found in this column). The darker colors emphasize low values, indicating genome pairs where only a small fraction of the genome was aligned (orange to white color gradient), or where there is a high difference in the length of the two genomes (black to white color gradient). The aligned genome fractions are expected to decrease with increasing the distance between the phages. Therefore, darker colors should correspond to genome pairs with low similarity values, and whiter colors to genome pairs with higher similarity values. Similarly, more closely-related phages are expected to have similar lengths. A 95% threshold was used for the species level demarcation and a 70% threshold for the genera level. According to the heatmap, Escherichia phage Ioannina belongs to the *Dhillonvirus* genus. The genome of the Escherichia phage Ioannina and the genome of other related dhillonviruses have 72.1 to 77.9 similarity.

SUPPLEMENTARY TABLES

Table S1. List of Escherichia phage Ioannina predicted CDSs, their positions on the phage genome, respective length, BLASTp annotation, putative role in phage life cycle and predicted protein size.

	Span			Length				
**CDSs**	**Start**	**Stop**	**Strand**	**CDS**	**aa**	**BLASTp annotation**	**Function in phage life cycle**	**Predicted Protein size (kDa)**
CDS1	1	573	+	573	190	Terminase small subunit	DNA recognition and initiation of DNA packaging	20,9
CDS2	573	1958	+	1386	461	Large terminase	DNA recognition and initiation of DNA packaging	52,2
CDS3	1971	3491	+	1521	506	Portal protein	Structure	55,4
CDS4	3469	4578	+	1110	369	Minor capsid protein	Structure	41,3
CDS5	4782	5012	+	231	76	Prokaryotic lipoprotein lipid attachment site	Packaging,host lysis	8,2
CDS6	5026	5787	-	762	253	Nucleotide modification associated domain 5	Modification function	28,6
CDS7	5841	6641	+	801	266	Minor structural protein	Structure	29,0
CDS8	6739	7860	+	1122	373	Major capsid protein	Structure	39,3
CDS9	7918	8418	+	501	166	Putative Head Tail Connector Protein	Packaging	18,2
CDS10	8421	8882	+	462	153	Head Tail Attachment	Packaging	17,0
CDS11	8974	9474	+	501	166	Tail component	Tail structure	18,5
CDS12	9476	9898	+	423	140	Tail completion protein	Tail structure	15,4
CDS13	9960	10685	+	726	241	Tail tube protein	Tail structure	25,7
CDS14	10699	11115	+	417	138	Tail assembly chaperone	Tail structure	15,9
CDS15	11137	11382	+	246	81	Major tail protein	Tail structure	9,1
CDS16	11586	11732	+	147	48	Hypothetical protein	Unknown function	5,2
CDS17	11784	11906	+	123	40	Hypothetical protein	Unknown function	4,3
CDS18	11833	14475	+	2643	880	Tail length tape measure protein	Tail structure	93,5
CDS19	14512	15111	+	600	199	Minor tail protein	Tail structure	21,7
CDS20	15108	15896	+	789	262	Minor tail protein L	Tail structure	28,8
CDS21	15899	16639	+	741	246	MPN family protein	Structure	27,7
CDS22	16591	17247	+	657	218	Tail assembly protein	Tail structure	22,5
CDS23	17244	20690	+	3447	1148	Tail protein	Tail structure	126,5
CDS24	20718	21683	-	966	321	Hypothetical protein	Unknown function	34,1
CDS25	21686	21910	-	225	74	Putative lipoprotein	Packaging,host lysis	7,8
CDS26	21992	24016	+	2025	674	Tail fiber protein	Tail structure	71,3
CDS27	24013	24270	+	258	85	Hypothetical protein	Unknown function	9,8

CDS28	24356	25084	+	729	242	Putative tail fiber protein	Tail structure	25,9
CDS29	25189	25764	-	576	191	Hypothetical protein	Unknown function	21,5
CDS30	25809	26279	-	471	156	DNA N-6-adenine-methyltransferase	Modification function	17,7
CDS31	26342	27034	-	693	230	Acid phosphatase	Modification function	26,5
CDS32	27015	27500	-	486	161	DNA N-6-adenine-methyltransferase	Modification regulation	18,8
CDS33	27487	27951	-	465	154	Hypothetical protein	Unknown function	17,7
CDS34	28034	28357	-	324	107	Putative HNH endonuclease	Modification regulation	11,8
CDS35	28354	29013	-	660	219	Cytosine specific methyltransferase	Modification regulation	24,9
CDS36	29067	30719	-	1653	550	Chromatin remodeling complex ATPase	Transcriptional function	62,4
CDS37	30755	30928	-	174	57	Putative kilA anti-repressor protein	Transcriptional function	6,9
CDS38	30968	31246	-	279	92	Nuclease	DNA replication	10,3
CDS39	31246	33534	-	2289	762	DNA polymerase I	DNA replication	86,2
CDS40	33531	33653	-	123	40	Hypothetical protein	Unknown function	4,6
CDS41	33643	34497	-	855	284	DNA helix destabilizing protein	DNA replication	32,0
CDS42	34579	34938	-	360	119	Mature oligodendrocyte transmembrane protein	Packaging	14,2
CDS43	34805	36235	-	1431	476	PD-(D/E)XK nuclease superfamily protein	DNA replication	52,4
CDS44	36235	36423	-	189	62	Putative regulatory protein binding pocket	Transcriptional function	7,2
CDS45	36423	36674	-	252	83	Hypothetical protein	Unknown function	9,9
CDS46	36723	37457	-	735	244	Hypothetical protein	Unknown function	27,7
CDS47	37478	37807	+	330	109	Helix-turn-helix domain protein	structural protein capable of binding DNA	12,4
CDS48	37820	40084	+	2265	754	Replicative DNA helicase	DNA replication	82,7
CDS49	40197	40583	-	387	128	Hypothetical protein	Unknown function	14,4
CDS50	40596	40901	+	306	101	Hypothetical protein	Unknown function	11,3
CDS51	40911	41180	+	270	89	Hypothetical protein	Unknown function	10,6
CDS52	41177	41773	+	597	198	Hypothetical protein	Unknown function	22,7
CDS53	41701	41910	+	210	69	Hypothetical protein	Unknown function	8,0
CDS54	41795	42019	-	225	74	Hypothetical protein	Unknown function	7,9
CDS55	41895	42182	+	288	95	Hypothetical protein	Unknown function	11,4
CDS56	42236	42538	+	303	100	Putative holin-like class II protein	Host lysis	10,5

CDS57	42535	42801	+	267	88	Putative holin-like class I protein	Host lysis	9,6
CDS58	42791	43276	+	486	161	Lysozyme	Host lysis	17,8
CDS59	43286	43693	+	408	135	Ig-like domain protein	Cell recognition	13,5
CDS60	43812	44954	+	1143	380	DNA polymerase II small subunit	DNA replication	42,6
CDS61	44951	45163	+	213	70	Hypothetical protein	Unknown function	8,0

Table S2. BLASTn alignment statistics of phage entries closely related to the Escherichia phage Ioannina identified in this study. Nucleotide identity full-length genome value represents the value of "Query coverage" (x) "Nucleotide identity" for each phage.

Scientific name	Accession number	Accession length	Query coverage	Nucleotide identity	Nucleotide identity full length genome
Siphoviridae sp. ctFRY1	BK032676.1	44490	89%	87.36%	77.75%
Escherichia phage Oekolampad	NC_073089.1	44882	83%	82.69%	68.63%
Sodalis phage SO1	NC_013600.1	45169	86%	82.80%	71.21%
Escherichia phage Gluttony_ev152	NC_073087.1	44825	88%	82.61%	72.70%
Escherichia phage BF9	NC_073083.1	44963	88%	82.37%	72.49%
Escherichia phage vB_EcoS_011D5	NC_073085.1	45204	85%	82.23%	69.90%
Escherichia phage vb_EcoS_bov25_1D	MT884015.2	44670	87%	82.06%	71.39%
Escherichia phage jat	NC_073076.1	44417	78%	83.57%	65.18%
Escherichia phage rolling	NC_073056.1	46017	85%	83.10%	70.64%
Escherichia phage mckay	NC_073073.1	44443	80%	82.95%	66.36%
Escherichia phage KarlBarth	NC_073057.1	43104	82%	82.78%	67.88%
Escherichia phage SECphi4	MT331608.1	44569	78%	82.65%	64.47%
Escherichia phage GeorgBuechner	NC_073066.1	44295	78%	82.49%	64.34%
Escherichia phage vB_Eco_Maverick	NC_073077.1	42624	80%	85.34%	68.27%
Escherichia phage vB_EcoS_L-h 1M	NC_073086.1	45204	82%	83.09%	68.13%

SUPPLEMENTARY FILES

Supplementary file S1. Genome organization synteny plot of Escherichia phage Ioannina compared to selected highly similar phages of the genus Dhillonvirus at the nucleotide level.

Supplementary file S2. MUSCLE (v3.8) amino acid sequence alignment of the putative tail fiber protein encoded by the isolated Escherichia phage Ioannina against phylogenetically closely related Escherichia phages.
