# Supplementary figures and images for: A Novel Dhillonvirus Phage against Escherichia coli Bearing a Unique Gene of Intergeneric Origin
Source: Curr Issues Mol Biol. 2024 Aug 23;46(9):9312–29. doi: 10.3390/cimb46090551 (PMC11430396; doi:10.3390/cimb46090551)

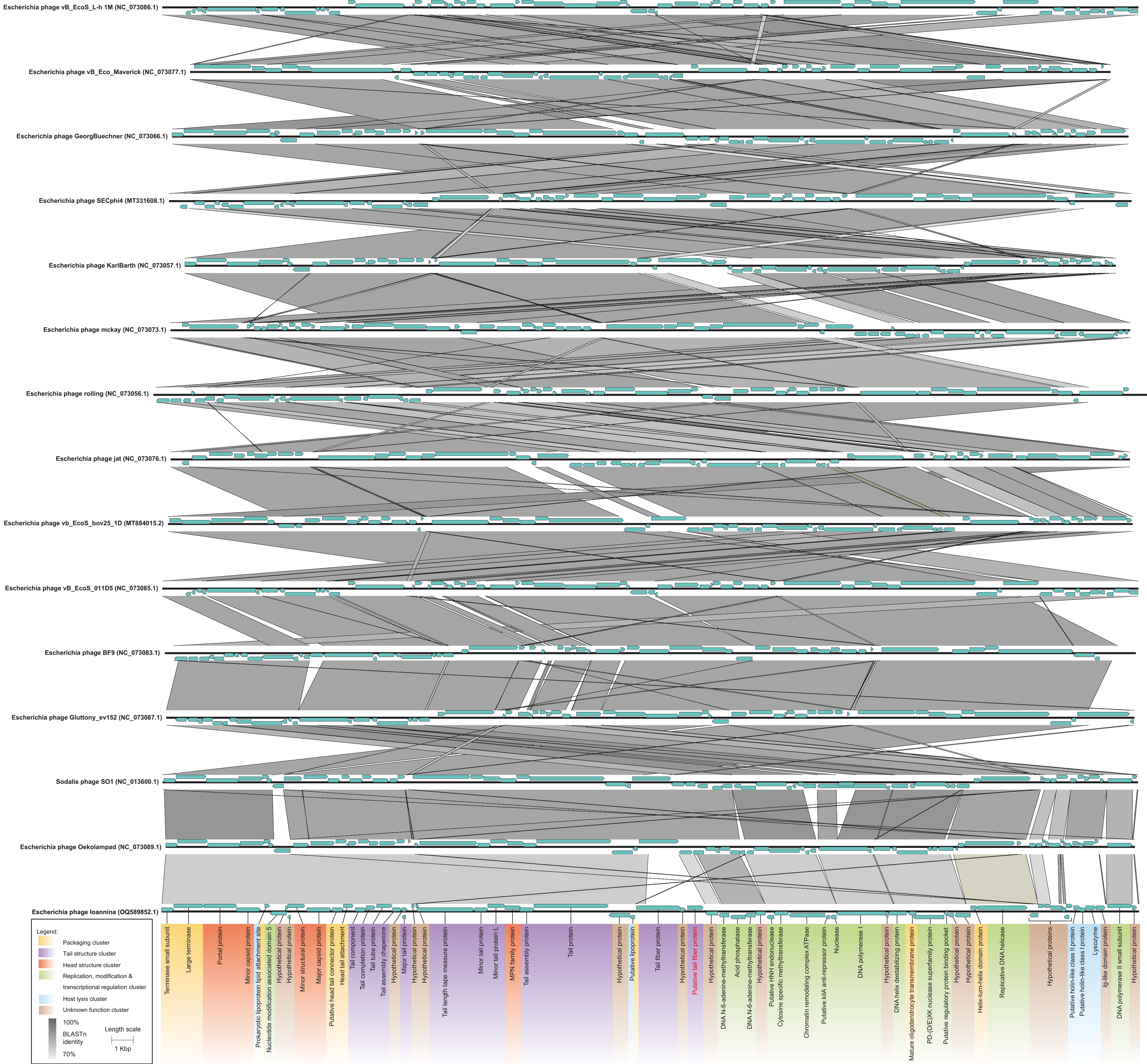

Supplement: Supplementary file 1 [file cimb-46-00551-s001.zip › Supplementary file S1.pdf]
